# Dual-targeting nanoparticles enhance microglial P2Y12R expression to promote neuronal mitophagy for repairing spinal cord injury

**DOI:** 10.1038/s41419-026-08596-2

**Published:** 2026-04-19

**Authors:** Zhenming Tian, Hong Li, Yunheng Jiang, Huiye Wei, Yubao Lu, Senyu Yao, Mao Pang, Xintao Shuai, Bin Liu, Limin Rong

**Affiliations:** 1 Department of Spine Surgery, The Third Affiliated Hospital of Sun Yat-Sen University, Guangzhou, China; 2 Guangdong Provincial Center for Quality Control of Minimally Invasive Spine Surgery, Guangzhou, China; 3 Guangdong Provincial Center for Engineering and Technology Research of Minimally Invasive Spine Surgery, Guangzhou, China; 4 Nanomedicine Research Center, the Third Affiliated Hospital of Sun Yat-sen University, Guangzhou, China

**Keywords:** Biomedical materials, Neuroimmunology, Cell death in the nervous system, Genetic vectors, Spinal cord injury

## Abstract

Spinal cord injury (SCI) leads to severe mitochondrial dysfunction and ROS cascade, with microglia playing a dual role in both exacerbating damage and providing neuroprotection. Recent evidence has highlighted the importance of P2Y12R in microglial-neuron interactions, particularly in modulating mitochondrial quality control and mitigating oxidative stress. Here, we develop a dual-targeting nanoparticle system (P2Y-TK-Nano) to enhance P2Y12R expression in microglia and promote neuronal mitophagy, aiming to reduce mitochondrial reactive oxygen species (mtROS) and improve neuronal survival following SCI. The P2Y-TK-Nano system combines a ROS-responsive thioketal bond for injury-site targeting with an MG1 peptide to selectively target microglia. This design enables precise nanoparticle delivery to the ROS-enriched injury microenvironment, effectively restoring P2Y12R expression in microglia. Microglia treated with P2Y-TK-Nano exhibit elevated P2Y12R expression, leading to increased interaction with injured neurons, improved mitophagy, and reduced mtROS production. These combined effects significantly attenuate secondary damage and contribute to neuroprotection post-SCI. Our findings reveal a novel regulatory mechanism by which P2Y12R overexpression in microglia enhances neuronal mitophagy and mitigates oxidative stress after SCI. The dual-targeting P2Y-TK-Nano system offers a promising therapeutic approach to address microglial activation and mitochondrial dysfunction in the context of SCI.

## Introduction

Spinal cord injury (SCI) causes severe secondary damage, leading to extensive neuronal loss and functional impairment. During this process, the injured tissue releases significant amounts of purines and reactive oxygen species (ROS), which act as key mediators of the injury response, exacerbating local inflammation and apoptosis [1]. Adenosine triphosphate (ATP), a central purinergic signaling molecule, plays a dual role in energy transfer within cells and as a damage-associated molecular pattern (DAMP) in the context of cellular injury, aggravating neurological outcomes [2, 3]. Extracellular ATP accelerates the progression from primary to secondary injury by amplifying neuroinflammation and driving a surge in ROS levels [4]. Neurons are particularly vulnerable to ROS, and under physiological conditions, they primarily rely on glycolysis in the somatic region to limit the production of mitochondrial ROS (mtROS) [5]. However, excessive ROS can damage mitochondria, triggering the release of mtROS and initiating a catastrophic “ROS-induced ROS release” (RIRR) cascade, which contributes to widespread cellular damage [6, 7]. While pharmacological interventions have been developed to scavenge exogenous ROS, targeted therapies for mtROS generated by mitochondrial injury remain insufficient. Mitophagy, an essential mitochondrial quality control (MQC)mechanism, plays a critical role in reducing mtROS by selectively eliminating damaged mitochondria [7]. However, excessive or unregulated mitophagy can also lead to cell death, underscoring the need for a “smart” regulator that can accurately target neurons with mitochondrial damage and activate mitophagy.

Microglia, the resident immune cells of the central nervous system (CNS), are pivotal in maintaining homeostasis and facilitating tissue repair following injury. These cells continuously monitor their microenvironment through dynamic interactions with neurons [8, 9]. Recent research has shown that neurons communicate their mitochondrial status to microglia via ATP release, which is detected by microglial P2Y12 receptors [10, 11]. This interaction promotes the formation of neuron-microglia junctions, enabling microglia to assess neuronal activity and mitochondrial function. In turn, microglia can modulate neuronal activity and mitochondrial integrity by releasing various regulatory molecules [12].

While extracellular ATP is detrimental in SCI, its degradation product, adenosine, exhibits neuroprotective properties in various CNS disorders [13–17]. However, adenosine’s short half-life limits its effectiveness as a sustained protective agent against prolonged pathological states [18]. Studies have shown that microglia can degrade extracellular ATP to adenosine through the activity of CD39 and CD73, thus providing neuroprotection [19]. Despite this, our previous research has demonstrated that P2Y12 receptor expression in microglia is downregulated following SCI, impairing their neuroprotective function [20]. This downregulation may hinder microglia’s ability to sense neuronal mitochondrial distress, resulting in the accumulation of ATP at the injury site and amplifying ROS-induced damage. Therefore, a strategy that selectively targets microglia to restore P2Y12R expression is critical for reactivating their neuroprotective capabilities.

In response to these challenges, our study developed a dual-targeting nanoparticle system incorporating a thioketal (TK) bond and MG1 peptide to specifically target microglia in the injury site and enhance P2Y12 receptor expression. Specifically, we employ polyethyleneimine (PEI) to deliver plasmids that promote P2Y12R overexpression in microglia [21, 22]. The TK bond enables the nanoparticle to target release PEI nanoparticles in the injury zone through ROS responsiveness and the MG1 peptide allows the accurate microglia recognition [23–25]. By upregulating P2Y12R expression, microglia can effectively degrade ATP to adenosine, thereby promoting mitophagy and reducing mtROS production. Ultimately, our research presents a novel mechanism of microglia-neuron interaction post-SCI and proposes a promising therapeutic strategy to target extracellular and intracellular ROS, as well as ATP accumulation.

## Results

### Synthesis and characterization of P2Y-TK-nano

**Fig. 1:**
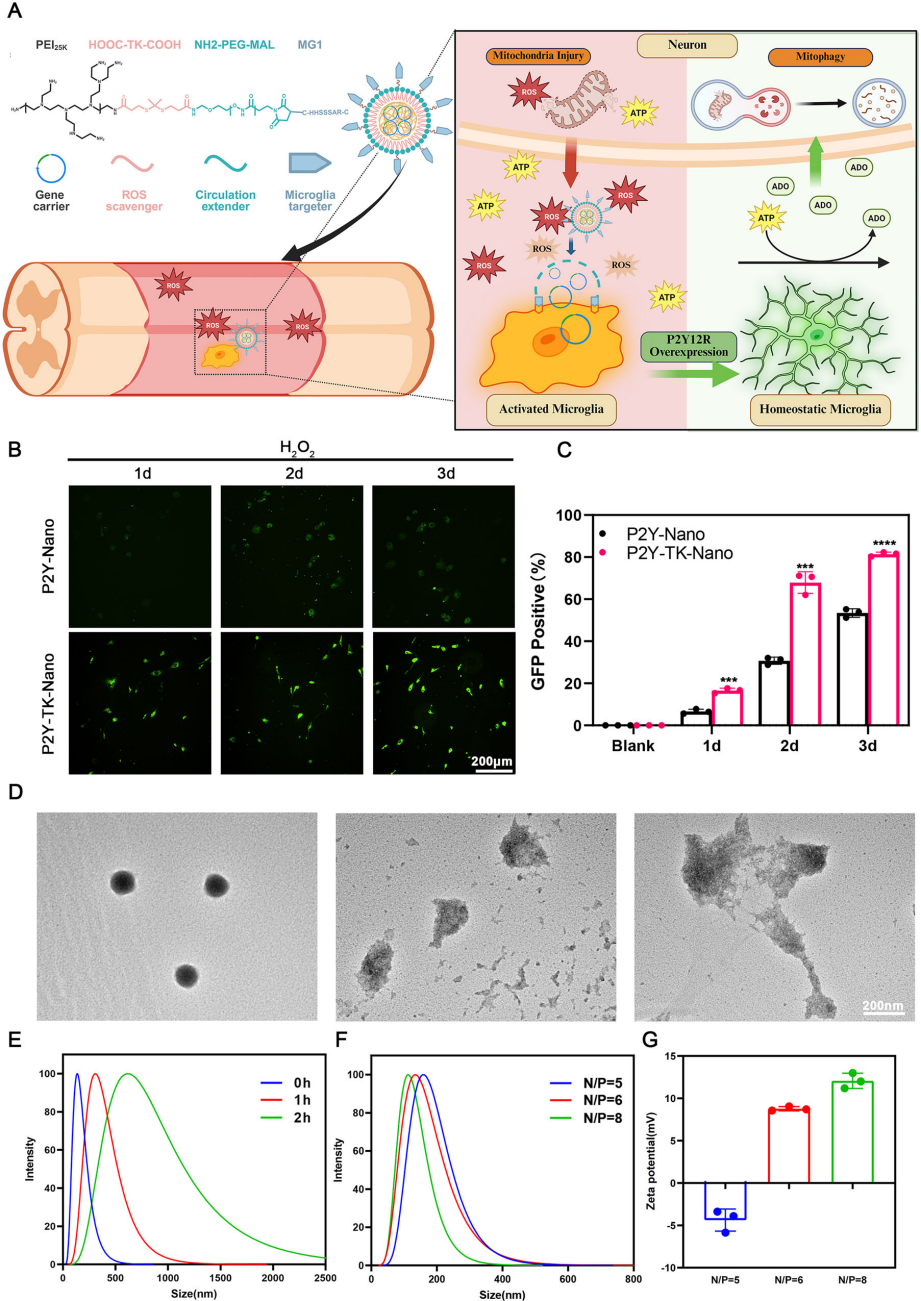
Evaluation of P2Y-TK-Nano gene delivery system design and ROS responsiveness. **A** Schematic diagram illustrating the construction of P2Y-TK-Nano and its protective mechanisms after SCI; **B** Immunofluorescence images showing P2Y-TK-Nano–mediated transfection of microglia in ROS-rich environments; **C** Percentage of GFP-positive microglia following P2Y-TK-Nano treatment (samples, *n* = 3); **D** TEM images depicting the ultrastructural morphology of P2Y-TK-Nano; **E** Particle size analysis of P2Y-TK-Nano in ROS-rich environments; **F** Particle size distribution of P2Y-TK-Nano of different N/P ratio; **G** Zeta potential measurements of P2Y-TK-Nano (samples, *n* = 3). Data are presented as mean ± SD. Statistical analysis was performed using one-way ANOVA; ***P < 0.001,****P  < 0.0001.

The P2Y12R gene plasmid was encapsulated within PEI for gene regulation. To target microglia, we conjugated the MG1 peptide to the PEG segment and designed ROS-sensitive TK bonds to encapsulate the gene carrier (Fig. 1A). Immunofluorescence confirmed that the PEG segment protects nanoparticles from phagocytosis, while in ROS-rich conditions, the P2Y-TK-Nano degrade through TK bond and release PEI for cell transfection (Fig. 1B, C, Supplementary Fig. 2D, E). Through optimization, we synthesized P2Y-TK-Nano with a size of approximately 132 nm and near-neutral surface potentials (Fig. 1F, G). Transmission electron microscopy (TEM) images showed that nanoparticles exhibited spherical morphology under normal conditions. Testing the responsiveness to ROS, the P2Y-TK-Nano displayed rapid expansion and collapse upon H₂O₂ exposure, indicating sensitivity to ROS in SCI lesions [26] (Fig. 1D, E). In contrast, the TK-free counterpart (P2Y-Nano) showed no discernible change under the same ROS conditions (Supplementary Fig. 1A, B). Further findings underscore P2Y-TK-Nano’s cytocompatibility and antioxidative capacity, highlighting its potential to shield cells from ROS-induced damage (Supplementary Fig. 2G, H).

### Downregulation of P2Y12R expression in microglia post-SCI

**Fig. 2:**
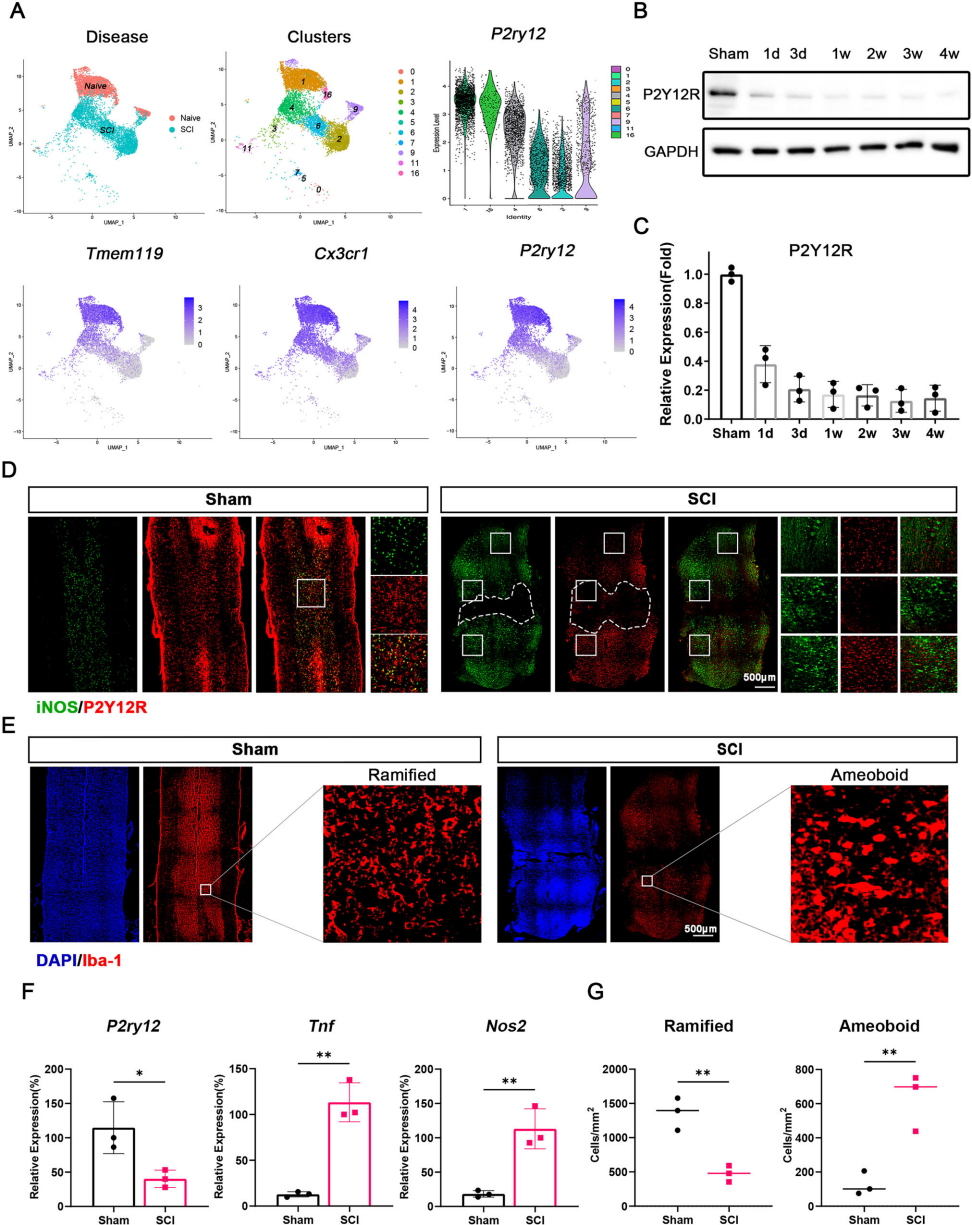
Downregulation of P2Y12R expression in microglia following spinal cord injury. **A** Single-cell sequencing results showing distinct clustering of spinal cord microglia at 7 days post-SCI **B** Western blot analysis displaying P2Y12R expression levels in microglia at various time points post-SCI; **C** Quantitative analysis of the expression of P2Y12R in (**B**) (mice, *n* = 3); **D** The immunofluorescence staining of spinal cords 1 week after injury, active microglia and homeostatic microglia were labeled with iNOS and P2Y12R respectively; **E** The immunofluorescence staining of spinal cords 1 week after injury, microglia were labeled with Iba-1; **F** Expression levels of active and homeostatic markers in spinal cord (mice, *n* = 3); **G** Quantitative analysis of the ratio of Ramified and amoeboid microglia in (**E**) (sections, *n* = 3). Data were presented as mean ± SD. Results were analyzed by Student’s t-test and One-way ANOVA. Significance: *P < 0.05, **P < 0.01.

Single-cell sequencing of spinal cord microglia at 7 days post-injury identified 16 clusters, including six microglial clusters (1, 16, 4, 6, 2, and 9). Clusters 1 and 16 represented normal microglia, while clusters 2, 4, and 6 were associated with injury-related microglia; cluster 9 showed transitional characteristics. P2Y12R expression was significantly downregulated in injured microglia (Fig. 2A), a finding further validated by Western blot analysis across various time points post-SCI, which is consistent with the findings of previous studies [27, 28] (Fig. 2B, C). Immunofluorescence revealed that P2Y12R expression was concentrated in homeostatic microglia, with reduced levels in activated microglia, which adopted an amoeboid morphology after SCI (Fig. 2D–G).

### P2Y12 plasmid transfection promotes microglial homeostatic state

To evaluate P2Y12R’s influence on microglial phenotype, H₂O₂-activated microglia were transfected with P2ry12-Nano (a non-PEG formulation lacking phagocytosis protection). P2Y12R expression, initially downregulated by H₂O₂ treatment, was restored upon P2ry12-Nano transfection, along with a reversion to a ramified morphology and a reduction in pro-inflammatory cytokine levels (Supplementary Fig. 2A–C). These findings suggest that P2Y12R upregulation fosters a homeostatic phenotype in microglia.

### P2Y-TK-nano’s role in protecting neurons from oxidative stress

The effect of P2Y-TK-Nano on neurons under oxidative stress was investigated by pre-treating microglia with P2ry12-Nano, followed by co-culture with H₂O₂-exposed neurons. Neuronal viability improved significantly in co-culture, with P2Y12R inhibition attenuating this protective effect (Supplementary Fig. 3A, C). Further analyses demonstrated that microglia overexpressing P2Y12R effectively reduced neuronal ROS levels (Supplementary Fig. 2B, D). Direct application of P2Y-TK-Nano and TK-Nano(empty nanocarriers) to neurons under oxidative stress yielded similar protective effects, confirming that while ROS response alone can protect neurons from oxidative damage, P2Y12R overexpression in neurons shows no protective effect (Supplementary Fig. 4).

### Enhanced mitochondrial quality control in neurons via P2Y12R-positive microglia

**Fig. 3:**
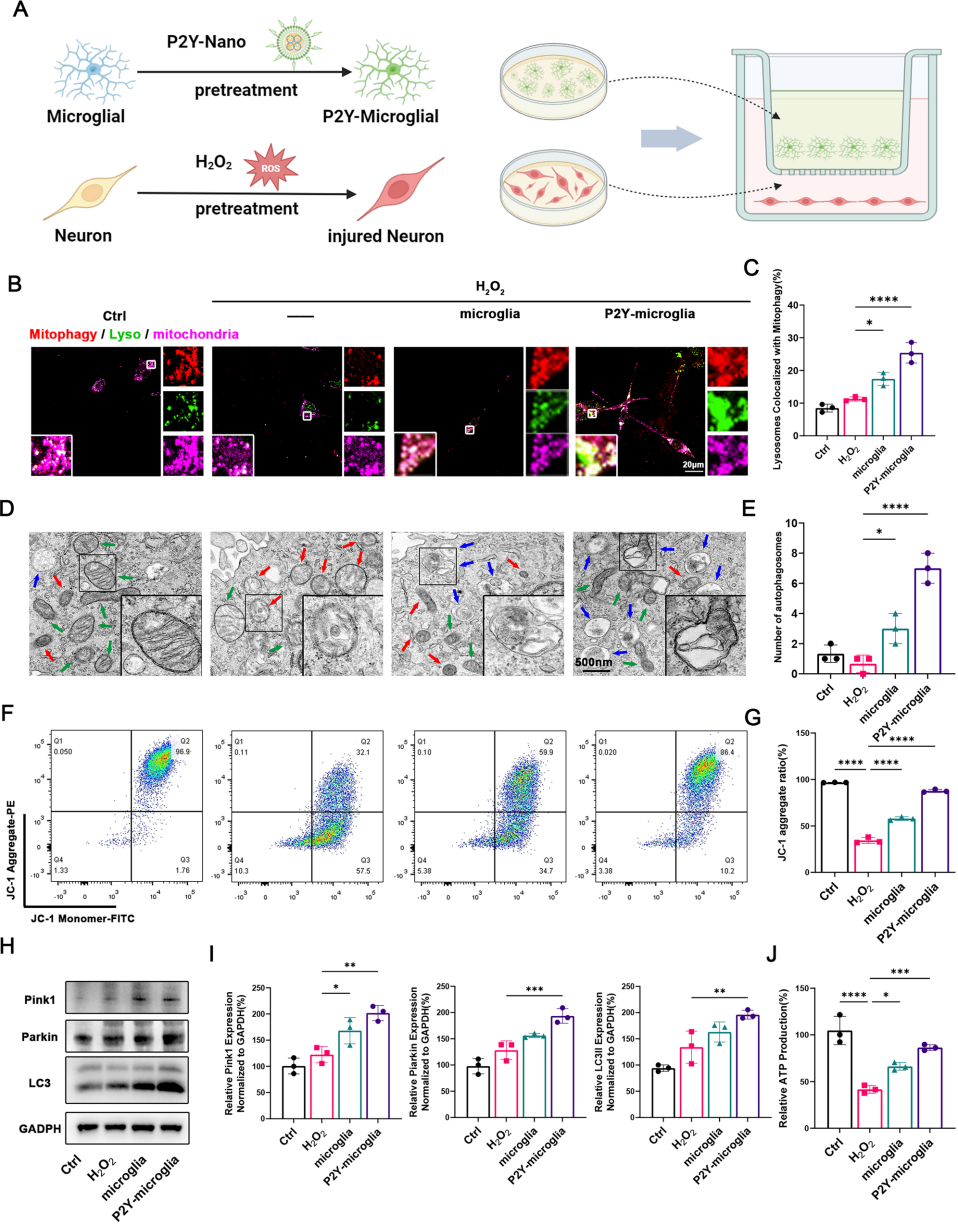
Role of P2Y12R-positive microglia in enhancing mitochondrial quality control in neurons. **A** Schematic diagram illustrating the co-culture model of neurons and microglia; **B** immunofluorescence of HT22 cells, lysosome, mitophagy, and mitochondria were labeled with Lyso Dye, Mitophagy and, Mitotracker respectively; **C** Quantification of the colocalization between lysosomes and mitophagy in (**A**) (sections, *n* = 3); **D** Representative TEM images of mitochondrial morphology in HT22 cells; **E** Quantitative analysis of the number of autophagosomes in (**D**) (sections, *n* = 3); **F** Flow cytometry analysis of MMP probed with JC-1 in HT22 cells; **G** Quantitative analysis of the ratio of JC-1 aggregates (PE channel) /JC-1 monomers FITC channel) in (**F**) (samples, *n* = 3); **H** Western blot of Pink1/Parkin /LC3 pathway; **I** Quantitative analysis of the expression of mitophagy related proteins in (**H**) (samples, *n* = 3); **J** Quantitative analysis of ATP production in HT22(samples, *n* = 3). Data were presented as mean ± SD. Results were analyzed by One-way ANOVA. Significance: *P < 0.05, **P < 0.01, ***P < 0.001, ****P  < 0.0001.

To investigate MQC, we measured Mitophagy in HT22 neurons co-cultured with P2ry12-Nano pre-treated microglia(P2Y-microglia). Mitophagy was significantly elevated in the co-cultured conditions, further enhanced by P2ry12-Nano treatment (Fig. 3B, C), and associated with increased autophagosomes within neurons (Fig. 3D, E). Additionally, P2Y12R overexpression restored mitochondrial membrane potential reduced by H₂O₂ (Fig. 3F, G). Western blot analyses showed that microglial P2Y12R overexpression enhanced PINK1/Parkin/LC3–mediated mitophagy in injured neurons and improved neuronal bioenergetics (Fig. 3H–J).

### Microglial degradation of ATP to ADO promotes neuronal mitophagy via the Pink1/Parkin pathway

**Fig. 4:**
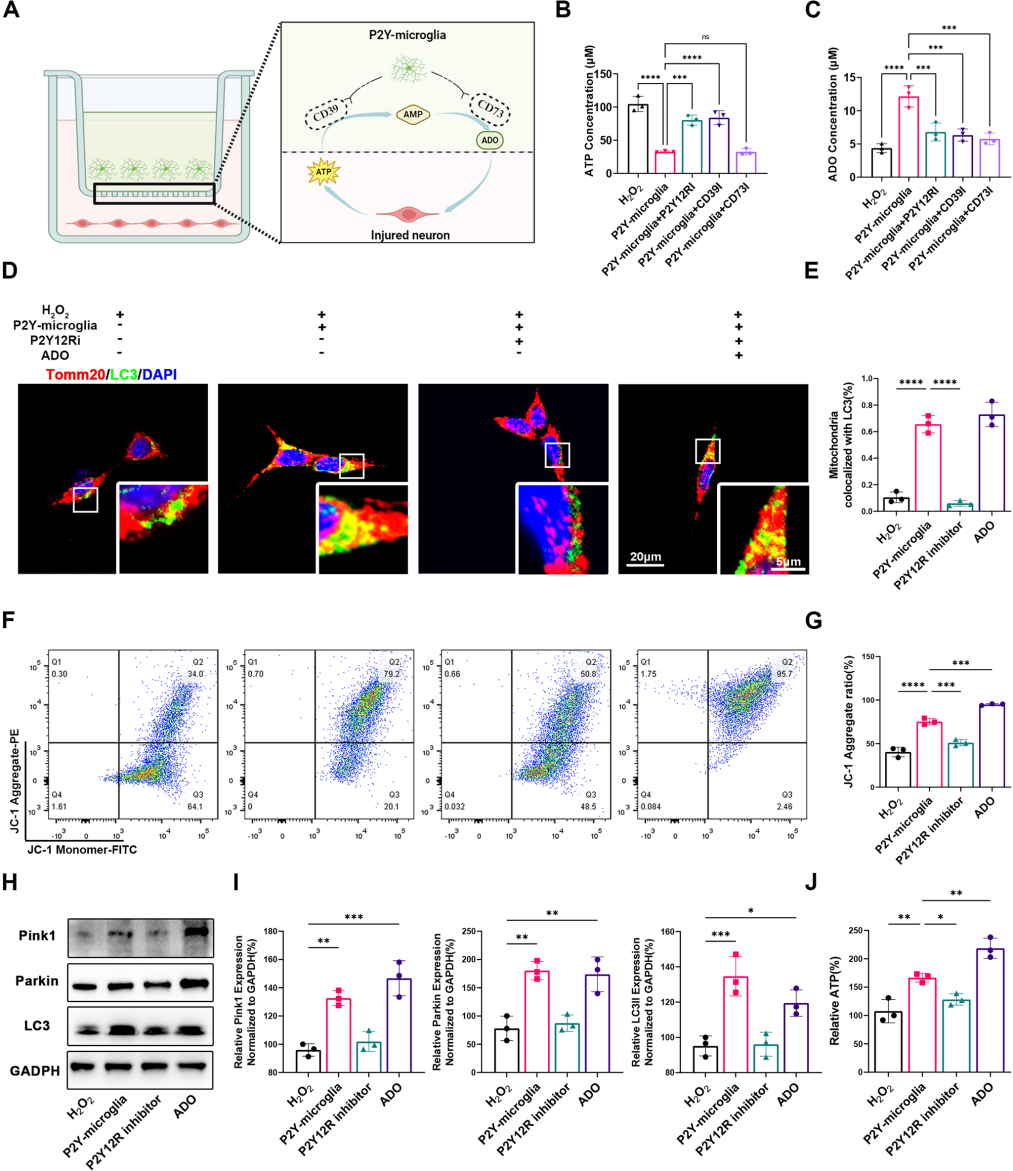
Microglial degradation of ATP to ADO enhances neuronal mitophagy. **A** Schematic representation of ATP recognition by microglia and its degradation to ADO through CD39 and CD79; **B**,**C** Quantitative analysis of the ATP and ADO concentration (samples, *n* = 3); **D** Immunofluorescence of HT22 cells, lysosome and mitochondria were labeled with LC3 and Tomm20 respectively; **E** Quantification of the colocalization between Tomm20 and LC3 in (**D**) (sections, *n* = 3); **F** Flow cytometry analysis of MMP probed with JC-1 in HT22 cells; **G** Quantitative analysis of the ratio of JC-1 aggregates (PE channel) /JC-1 monomers FITC channel) in (**F**) (samples, *n* = 3); **H** Western blot of Pink1/Parkin /LC3 pathway; **I** Quantitative analysis of the expression of mitophagy related proteins in (**H**) (samples, *n* = 3); **J** Quantitative analysis of ATP production in HT22(samples, *n* = 3). Data were presented as mean ± SD. Results were analyzed by One-way ANOVA. Significance: *P < 0.05, **P < 0.01, ***P < 0.001, ****P < 0.0001, ns means no significance.

Studies indicate that microglia can recognize ATP via P2Y12R and subsequently degrade it to adenosine (ADO) (Fig. 4A) [19]. Our findings demonstrate that blocking P2Y12R, CD39, and CD73 effectively inhibits the ability of microglia to degrade ATP into ADO (Fig. 4A–C, Supplementary Fig. 5A–C). To explore the role of microglial mediated ATP degradation in neuronal mitophagy, we examined mitochondrial autophagy and energy metabolism using immunofluorescence (Fig. 4D, E), mitochondrial membrane potential (Fig. 4F, G) and ATP assays (Fig. 4J). H₂O₂ treatment mildly activated neuronal mitophagy, which was significantly enhanced by adding P2Y-microglia, a process inhibited by a P2Y12R antagonist, indicating P2Y12R dependence. Addition of ADO reactivated mitophagy, implying ADO-dependent activation via adenosine receptors (AR). Western blot analyses demonstrated that P2Y12R-overexpressing microglia promoted mitophagy in injured neurons by activating the PINK1/Parkin/LC3 pathway (Fig. 4H, I).

To pinpoint the AR subtype involved, ADO-treated neurons were exposed to specific AR inhibitors. Blocking A1R significantly suppressed mitophagy, while A2AR and A2BR inhibition had no effect (Supplementary Fig. 6B). Pharmacological blockade of the adenosine A1 receptor (A1R) prevented P2Y12R-overexpressing microglia from activating the PINK1/Parkin/LC3 mitophagy pathway (Supplementary Fig. 5D–F). Consistently, siRNA-mediated knockdown of PINK1 or Parkin disrupted ADO-induced mitophagy (Supplementary Fig. 6C–F). These results suggest that microglia use P2Y12R to recognize neuron-released ATP, degrade it to ADO, and subsequently activate neuronal mitophagy through A1R.

### Microglia P2Y12R mediates neuron protection through microglia-neuron-astrocyte crosstalk

Given the tight interplay among astrocytes, microglia, and neurons in the CNS, we examined P2Y12R-dependent cross-talk in a neuronal injury model. In neuron–astrocyte co-cultures, basal extracellular ATP concentration in the culture medium did not increase. Under injury conditions, however, ATP concentration rose markedly in the astrocyte co-culture condition yet did not improve neuronal bioenergetics (Supplementary Fig. 7B, C). Introducing microglia to form tri-cultures under the same paradigm significantly reduced ATP in the medium and produced a pronounced elevation of ADO. Pharmacological blockade of P2Y12R restored ATP accumulation and returned ADO to baseline, consistent with microglial P2Y12R sensing of neuron-derived ATP and its sequential hydrolysis by CD39/CD73 to ADO (Supplementary Fig. 7E-G). This trend was also consistent in the validation of mitophagy-related outcomes (Supplementary Fig. 7D, H, I).Together, we propose a working model in which neuron-driven extracellular ATP transients are amplified and by astrocytes and then decoded by microglia via P2Y12R; through CD39/CD73-dependent purine metabolism, microglia convert ATP into adenosine, which in turn exerts a protective, mitophagy-promoting effect on injured neurons.

### P2Y-TK-nano promotes mitophagy in ex vivo spinal cord slices

Using an ex vivo organotypic spinal cord slice model, we investigated P2Y-TK-Nano’s modulation of microglia-mediated neuroprotective effects on neurons (Supplementary Fig. 8A) [29]. FITC-labeled P2Y-TK-Nano demonstrated MG1 receptor-mediated microglial targeting specificity with minimal off-target binding to other cell types. Under non-ROS conditions, reduced co-localization with microglia was observed, whereas ROS-rich injury microenvironments triggered spatiotemporal nanoparticle activation, enhancing targeted delivery to microglial cells (Supplementary Fig. 8B–D). TUNEL staining demonstrated P2Y-TK-Nano’s neuroprotective effects within spinal cord tissue. Co-staining with TUNEL and NeuN revealed reduced neuronal apoptosis in H₂O₂-injured slices treated with P2Y-TK-Nano (Supplementary Fig. 9A–D). Enhanced mitophagy was evidenced by increased co-localization of Tomm20 and LC3 in neurons treated with P2Y-TK-Nano (Supplementary Fig. 9E–G), suggesting that P2Y-TK-Nano promotes mitophagy in residual neurons following SCI.

### P2Y-TK-nano targets microglia in the injured spinal cord

To evaluate P2Y-TK-Nano accumulation in the injured spinal cord, we injected nanoparticles intravenously in both normal and SCI mice and tracked fluorescence in spinal cord and visceral organs at various time points (Supplementary Fig. 10A, B). In SCI mice, fluorescence appeared in the spinal cord within 4 hours, peaked at 12 hours, and decreased by 24 hours (Supplementary Fig. 10C). Comparisons across nanoparticle formulations revealed that PEG-TK-MG1-PEI (P2Y-TK-Nano) exhibited the highest accumulation at the injury site, while PEG-PEI showed minimal accumulation due to its lack of targeting and ROS-responsive features (Supplementary Fig. 10D, E). Further analysis with FITC-labeled P2Y-TK-Nano showed significant co-localization with microglia in the injury site. Without the MG1 peptide, co-localization decreased notably, and microglia displayed an activated ameboid morphology, suggesting P2Y-TK-Nano’s capability to penetrate the blood-spinal cord barrier (BSCB) and target microglia (Supplementary Fig. 10F, G).

### P2Y-TK-nano alleviates early microglial activation in SCI

**Fig. 5:**
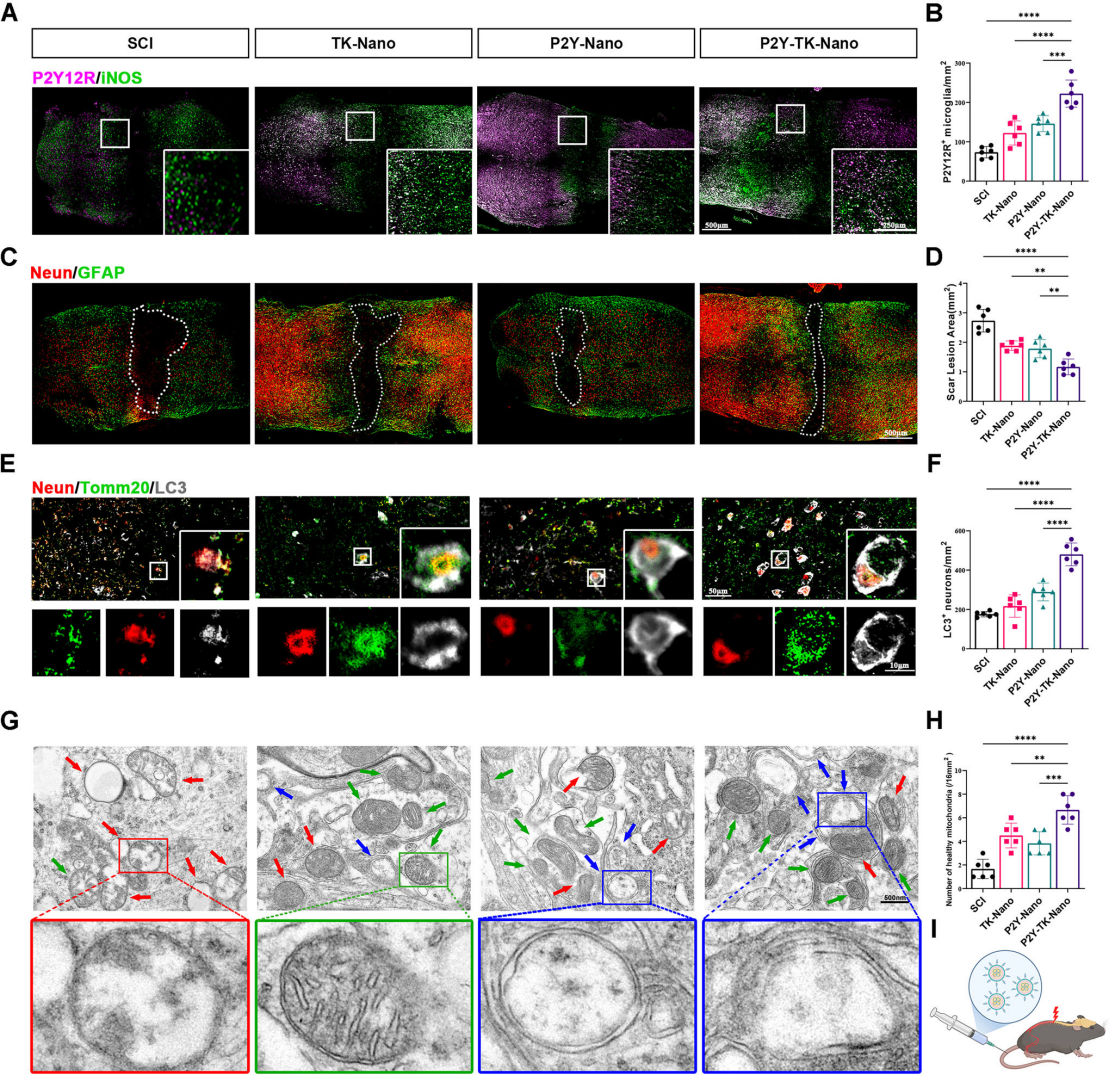
Effects of P2Y-TK-Nano on microglial activation and mitophagy in neurons 1-week post-SCI. **A** The immunofluorescence staining of spinal cords, active microglia and homeostatic microglia were labeled with iNOS and P2Y12R respectively; **B** Quantification of the P2Y12R positive microglia in (**A**) (mice, *n* = 6); **C** The immunofluorescence staining of spinal cords, neuron and astrocytes were labeled with Neun and GFAP, respectively; **D** Quantification of the lesion area in (**C**) (mice, *n* = 6); **E** The immunofluorescence staining of spinal cords, autophagic cells, mitochondria, and neurons were labeled with LC3, Tomm20, and Neun, respectively; **F** Quantification of the LC3 positive neuron in (**E**) (mice, *n* = 6); **G** Representative TEM images of mitochondrial morphology in lesion area, **H** Quantitative analysis of the number of healthy mitochondria in (**G**) (sections, **n** = 6); **I** Schematic representation of the P2Y-TK-Nano injection for SCI repair. Data were presented as mean ± SD. Results were analyzed by One-way ANOVA. Significance: **P < 0.01, ***P < 0.001, ****P < 0.0001.

To assess microglial activation following SCI, TK-Nano or P2Y-TK-Nano were injected into SCI mice, and spinal cord sections were analyzed seven days post-injury. In uninjured spinal cords, microglia exhibited a homeostatic state with high P2Y12R expression and low iNOS levels. After injury, P2Y12R expression decreased while iNOS levels increased, indicating microglial activation. TK-Nano suppressed this activation by lowering ROS. Although P2Y-Nano directly modulates microglial gene expression, its effect was comparable to TK-Nano, likely because the absence of a thioketal linker reduced ROS-triggered release within lesions and the ROS-rich milieu remained unfavorable for reversion to a homeostatic state. P2Y-TK-Nano further restored microglia to a homeostatic state, as indicated by the elevated P2Y12R expression, underscoring the importance of combining genetic regulation with environmental modification. (Fig. 5A, B).

### P2Y-TK-nano enhances mitochondrial autophagy in neurons Post-SCI

P2Y-TK-Nano’s neuroprotective effects were examined by staining for neuronal (Neun), astrocytes (GFAP), and mitochondrial autophagy (Tomm20, LC3) markers in spinal cords seven days post-injury. P2Y-TK-Nano significantly reduced the lesion cavity size and enhanced mitochondrial autophagy in neurons compared to SCI, P2Y-Nano and TK-Nano groups (Fig. 5C–F). Electron microscopy confirmed that P2Y-TK-Nano promoted the formation of autophagosomes and increased the number of healthy mitochondria, indicating enhanced mitophagy (Fig. 5G, H).

### P2Y-TK-nano facilitates nerve regeneration in the subacute phase of spinal cord injury

We next assessed subacute therapeutic effects by performing transmission electron microscopy and immunohistochemistry on spinal cord sections at 4 week post-SCI. The SCI group exhibited the highest g-ratio, consistent with demyelination, whereas both TK-Nano and P2Y-Nano showed partial normalization. P2Y-TK-Nano treatment resulted in the most robust regeneration, with well-formed myelin (Supplementary Fig. 11A, E, F). Masson staining revealed fewer cavities in the treatment groups compared to the SCI group (Supplementary Fig. 11B). Immunofluorescence staining for NF200 (neurofilament) and MBP (myelin) further demonstrated reduced nerve damage in the P2Y-TK-Nano group, suggesting that overexpression of P2Y12R enhances neuronal survival and axonal regrowth (Supplementary Fig. 11C, D). GFAP and TUJ1 staining indicated that glial scar formation was smaller in both nanoparticle-treated groups compared to the SCI group. However, the neurofilament-labeled damage area was larger than the glial scar, emphasizing the need for early neuronal protection (Supplementary Fig. 11G, H).

### P2Y-TK-nano promotes behavioral recovery and neural circuit rebuilding in the chronic phase of spinal cord injury

**Fig. 6:**
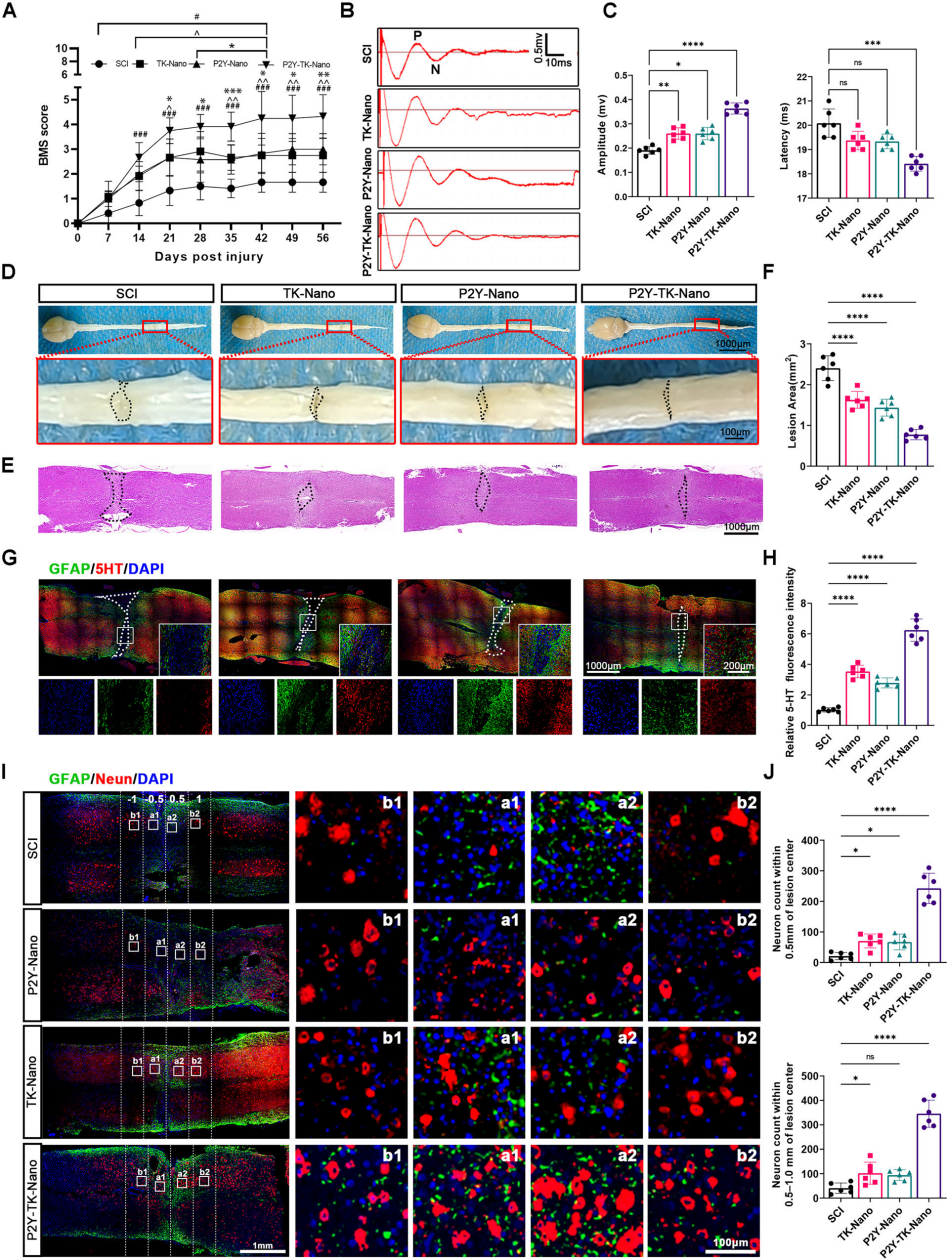
Assessment of nerve regeneration and functional recovery 8-weeks post-SCI with P2Y-TK-Nano treatment. **A** The BMS score of the hindlimb of SCI mice; **B** Evoked potentials of the sciatic nerve of the left hindlimb in mice at 8 weeks post-injury; **C** Statistics of the amplitude and latency of Evoked potentials in (**B**) (mice, *n* = 6); **D** Representative gross images of the brain and spinal cord following SCI; **E** HE staining of the spinal cords in each group; **F** Quantification of the lesion area in (**E**) (mice, *n* = 6); **G** The immunofluorescence staining of spinal cords, axons and astrocytes were labeled with 5-HT and GFAP respectively; **H** Quantification of the 5-HT fluorescence intensity in (**G**) (mice, *n* = 6); **I** The immunofluorescence staining of spinal cords, neuron and astrocytes were labeled with Neun and GFAP, respectively; **J** Quantification of the neuron count in (**I**) (mice, *n* = 6). Data were presented as mean ± SD. Results were analyzed by One-way ANOVA. Significance: *P < 0.05, **P < 0.01, ***P < 0.001, ****P < 0.0001, ns means no significance.

Finally, we assessed chronic-phase outcomes at 8 weeks post-SCI in mice, integrating behavioral testing and histological analyses. Motor function recovery was assessed using BMS scores and neurophysiological measurements. Initial BMS scores were zero for all groups, confirming successful SCI induction. Over time, the P2Y-TK-Nano group exhibited superior recovery compared to the TK-Nano, P2Y-Nano and SCI groups. By day 21, the P2Y-TK-Nano group achieved significantly higher BMS scores than TK-Nano and P2Y-Nano. This advantage further widened by day 42 and then stabilized, remaining at a plateau from days 42 to 56 (Fig. 6A). MEP recordings also showed reduced latency in both P2Y-Nano and TK-Nano groups; however, only the P2Y-TK-Nano group significantly improved amplitude and latency, indicating better functional recovery (Fig. 6B, C).

Gross examination of spinal cords showed that P2Y-TK-Nano markedly reduced the lesion, leaving only a thin macroscopic scar line (Fig. 6D). Hematoxylin–eosin staining corroborated these changes, and lesion-area quantification confirmed modest reductions with TK-Nano and P2Y-Nano, with the smallest lesion in the P2Y-TK-Nano group (Fig. 6E, F). To evaluate circuit reconstruction, we stained 5-HT to visualize descending corticospinal tract projections. In the SCI group, the lesion gap was wide and virtually devoid of 5-HT; sparsely crossing fibers were occasionally observed after P2Y-Nano or TK-Nano. By contrast, P2Y-TK-Nano yielded dense 5-HT labeling spanning the lesion, accompanied by a less compact GFAP-defined glial border (Fig. 6G, H). Finally, NeuN analysis showed near-absence of neurons within 0.5 mm of the lesion core in SCI and only sparse neurons at 1 mm; P2Y-Nano and TK-Nano produced limited improvement, whereas P2Y-TK-Nano preserved neuronal density within 0.5 mm to levels comparable to more distal regions, mirroring the 5-HT pattern (Fig. 6I, J). These data indicate that P2Y-TK-Nano more effectively rebuilds neural circuits and supports functional recovery.

## Discussion

This study aimed to explore a novel therapeutic approach for SCI utilizing a dual-targeting nanoparticle system (P2Y-TK-Nano) to enhance microglial P2Y12R expression, promoting neuronal mitophagy and reducing mtROS release. Our results show that microglia overexpressing P2Y12R effectively interact with injured neurons by degrading released ATP to adenosine, thereby promoting mitophagy, improving MQC, and reducing oxidative stress. This work offers new insights into the therapeutic potential of microglia in SCI recovery and provides an effective tool for addressing mtROS-induced neuronal damage in the CNS.

After SCI, the disruption of the BSCB and infiltration of inflammatory cells result in elevated ROS levels, which are a major cause of secondary injury and cell death [1, 26]. MtROS, generated during mitochondrial oxidative phosphorylation, are key contributors to this oxidative stress. Uncontrolled mtROS release can trigger a massive ROS amplification known as the RIRR effect, exacerbating the damage. Mao et al. developed a mitochondrial-targeted mtROS scavenger (TSC) using superoxide dismutase and catalase nano-complexes, which reduces oxidative stress-related inflammation and pyroptosis [30]. However, these approaches fail to differentiate between healthy and damaged mitochondria, reducing therapeutic efficiency. Moreover, mtROS are essential biological signals that activate protective pathways such as mitophagy, which clears dysfunctional mitochondria [6]. Non-specific clearance of mtROS may interfere with these natural processes, leading to adverse effects. Thus, a more precise approach is needed to selectively target damaged mitochondria and trigger mitophagy without excessive mitochondrial clearance. Microglia are known to exacerbate neuroinflammation post-SCI by releasing pro-inflammatory factors, contributing to secondary injury. However, their neuroprotective role, particularly through purinergic receptors, has recently gained attention. After traumatic brain injury, P2Y6R, P2Y12R, and P2X4R mediate microglial morphology changes, contributing to glial scar formation [1]. P2Y6 activation regulates microglial engulfment of neuronal debris and cytokine production, while P2X7R is involved in inflammatory activation through the NLRP3 inflammasome [31, 32]. In the context of SCI, P2Y12R, a marker of homeostatic microglia, plays a crucial role in microglia-neuron interactions. It enables microglia to respond to ATP released from injured neurons and migrate toward the injury site, where they modulate neuronal recovery [33]. Recent studies have shown that microglia can sense neuronal mitochondrial activity via P2Y12R to assess neuronal health [34].

Our study demonstrated that SCI leads to microglial activation and a subsequent downregulation of P2Y12R. However, neonatal microglia can restore P2Y12R expression after SCI, a process associated with scar-free neural regeneration [27]. We extend this finding to adult models, showing that P2Y-TK-Nano treatment restored P2Y12R expression in activated microglia, allowing them to revert to a protective ramified morphology and enhancing their ability to promote neuronal mitophagy. This upregulation of P2Y12R not only restores microglial function but also reduces mtROS production in neurons, limiting secondary damage. The therapeutic advantage of P2Y-TK-Nano lies in its dual-targeting design. The nanoparticle system combines a ROS-responsive TK linkage with the microglia-targeting MG1 peptide, enabling precise targeting of microglia in the oxidative stress environment of the injury site. This specificity improves upon previous strategies that lacked the ability to distinguish between microglia and macrophage, which relied on phagocytosis or general surface markers like α4β1 integrin and intercellular adhesion molecule-1 [35–38]. The ROS-responsive TK linkage ensures selective release of the therapeutic agent in regions of high oxidative stress, minimizing off-target effects [23]. Additionally, the MG1 peptide targets microglia, which are most prevalent at the injury site, further enhancing therapeutic efficacy [39, 40]. Animal studies indicate that, in the acute phase after SCI, P2Y-TK-Nano significantly attenuated microglial activation within the lesion and reduced neuronal injury. By the subacute (4-week) and chronic (8-week) stages, these early neuroprotective effects translated into pronounced reconstruction of neural circuits. While P2Y12R modulation in microglia offers significant promise for SCI treatment, our findings are constrained to the specific mouse models and ex vivo systems studied. The broader applicability of this dual-targeting strategy to other CNS disorders remains to be validated in future studies.Furthermore, large animal studies that closely resemble human pathology will be critical for translating this therapy to clinical use.

Despite the promising results, some limitations must be acknowledged. Firstly, The MG1 peptide, used to target microglia, detaches with the PEG layer after TK cleavage. We have demonstrated both in vivo and ex vivo that P2Y-TK-Nano efficiently and specifically targets and transfects microglia in ROS-enriched environments. We propose that this specificity is due to the simultaneous ROS response and MG1-mediated targeting of microglia, which enables localized plasmid release via PEI for effective transfection. Future improvements could involve modifying the PEI layer with MG1 peptide to enhance microglial targeting after PEG detachment. Additionally, in this study we performed a preliminary exploration of the interactions among neurons, microglia, and astrocytes. At the level of ATP signaling, our data suggest that astrocytes act as “amplifiers” of neuron-derived ATP signals, whereas microglia function as ATP “decoders” via P2Y12R, converting ATP into adenosine through CD39 and CD73 to exert protective effects on injured neurons. We fully acknowledge, however, that the genuine tri-cellular crosstalk in the injured spinal cord is far more complex than this simplified model. Neurons, microglia, and astrocytes engage in complex interactions through “quad-partite” synapses, which are critical for regulating neural activity [41]. A more in-depth dissection of this tri-cellular axis will be a priority for our future studies.

## Materials and Methods

### Synthesis of COOH-TK-PEG5K-MG1

To synthesize COOH-TK-PEG5K-MG1, NH2-PEG5K-Mal (500 mg) was dissolved in 5 mL of 20 mM MES buffer (pH 7.0). MG1 peptide (250 mg) was then added, and the reaction mixture was stirred at room temperature for 24 h. The pH was adjusted to 6.5 with HCl, and 30 mg of EDC/NHS-activated TK-(CH2CH2COOH)2 was introduced. This reaction proceeded overnight at room temperature. The resulting solution was dialyzed using a 1000 MWCO dialysis bag against pure water for 24 h, with water replaced every 4 h. The final dialysate was freeze-dried, yielding COOH-TK-PEG5K-MG1 in powder form, which was characterized by NMR spectroscopy.

### Synthesis of PEI-TK-PEG-MG1

For PEI25k -TK-PEG5K-MG1 synthesis, COOH-TK-PEG5K-MG1(60 mg) was dissolved in 5 mL of 20 mM MES buffer (pH 6.5) with EDC (2 mg) and NHS (1.2 mg), stirred for 30 min. In parallel, PEI25k (250 mg) was dissolved in 5 mL of MES buffer, sonicated for 3 min, and the pH was adjusted to 6.5. The activated COOH-TK-PEG5K-MG1 solution was added to PEI25k, and the mixture reacted for 24 h at room temperature. The product was dialyzed using a 5000 MWCO dialysis bag for 24 h against pure water and then freeze-dried. The resulting PEI-TK-PEG-MG1 was characterized by NMR spectroscopy. FITC-labeling was achieved by overnight incubation of FITC with PEI-TK-PEG-MG1 in an aqueous solution.

### Synthesis and characterization of P2Y-TK-nano

PEI-TK-PEG-MG1 (52 mg) was dissolved in 20 mM Tris-HCl buffer (pH 6.5) to form a 0.01 M PEI solution. P2ry12 plasmid DNA (2 µg) was combined with 3, 3.6, or 4.8 µL of PEI solution, and Tris-HCl buffer was added to reach a final volume of 50 µL. The mixture was gently vortexed and left at room temperature for 30 min to form P2Y-TK-Nano at N/P ratios of 5, 6, and 8. P2Y-TK-Nano particles with an N/P ratio of 6 were dissolved in 100 nM H_2_O_2_ and incubated at room temperature. Particle size and morphology were monitored over time using dynamic light scattering and TEM with a JEOL JEM-2010HR. Hydrodynamic radius and zeta potential were analyzed using Zetasizer Nano ZS, and 1H NMR spectra were obtained with Varian Unity.

### In Vivo Tracking of Nanoparticles

Fluorescent tracking nanoparticles were prepared by mixing PEG-PEI, PEG-MG1-PEI, and PEG-TK-MG1-PEI with ICG (0.01 M) in a volume ratio of 6:1. After intravenous injection into normal and SCI-induced mice (2 h post-SCI), fluorescence was detected at 1, 4, 8, 12, and 24 h in the spinal cord and organs using an IVIS Lumina LT Series III imaging system (excitation: 780 nm, emission: 810 nm).

### Single-cell RNA sequencing of microglia post-SCI

Single-cell RNA sequencing of microglia was conducted using a previously published database (Series GSE198852) [20]. C57BL/6 female mice (8 weeks old) were acquired from the Guangdong Animal Experiment Center and maintained in SPF conditions. SCI was performed, and 7 days post-injury, myeloid cells were isolated for sequencing. The spinal cord was enzymatically dissociated into single-cell suspensions, enriched with CD11b microbeads. Libraries were prepared using the BD Rhapsody system and sequenced on an Illumina Novaseq 6000 PE150 platform.

### Isolation of primary microglia and astrocytes

Neonatal C57BL/6 mice (postnatal day 0-2, both sexes) were obtained from the Guangdong Animal Experiment Center and used immediately for experiments after arrival. Mice were euthanized by rapid decapitation under deep isoflurane anesthesia. Cortices were dissected, meninges removed, and enzymatically dissociated. The cell suspension was filtered through a 70 µm strainer and seeded on poly-D-lysine–coated flasks in DMEM/F12 with 10% serum to establish mixed glial cultures. Cultures were maintained at 37 °C/5% CO₂ with medium changes every 2–3 days. After reaching confluence, microglia were collected by a short orbital shake-off (200 rpm, 60 min) and the supernatant was harvested and replated. To enrich astrocytes, the original flasks were subjected to an extended overnight shake (200 rpm, 16 h) to remove residual OPCs and loosely adherent cells. The remaining adherent monolayer was gently detached with trypsin and replated. Purity was verified by immunostaining (microglia Iba1 /P2RY12-positive; astrocytes GFAP/S100β positive).

### Microglial transfection with P2Y-TK-nano

Primary microglia were seeded onto poly-L-lysine–coated coverslips. Cells were then incubated with P2Y-TK-Nano (GFP-tagged) or P2Y-Nano (GFP-tagged) in medium containing 100 μM H₂O₂ to mimic oxidative stress conditions for 12 h. After incubation, microglia were washed three times with warm PBS and maintained in fresh complete medium. GFP expression was monitored at days 1, 2, and 3 using a laser scanning confocal microscope.

For quantification of transfection efficiency, GFP-positive microglia were counted in 3 randomly selected fields per coverslip, and the total number of nuclei was determined by DAPI staining. The percentage of transfected cells was calculated using ImageJ software. Data are from at least three independent experiments.

### Cell co-culture experiments

For co-culture experiments, primary microglia, neurons, and astrocytes were maintained in a Transwell system. Microglia and/or astrocytes were seeded in the upper insert, while neurons were plated in the lower chamber to prevent direct cell–cell contact. At the indicated time points, the inserts containing microglia and/or astrocytes were removed, and all analyses were performed directly on the neurons in the lower chamber. For co-culture experiments, primary microglia, neurons, and astrocytes were maintained in a Transwell system (0.4 µm pore size inserts, Corning). Microglia and/or astrocytes were seeded in the upper inserts at a density of 1 × 10^5 cells/insert, and neurons were plated in the lower chamber at a density of 2 × 10^5 cells/well to prevent direct cell–cell contact. Cells were allowed for pretreatment for 24 h before the start of treatments. At the indicated time points, the inserts containing microglia and/or astrocytes were carefully removed, and all readouts were obtained directly from the neurons and/or culture medium in the lower chamber.

For imaging-based quantification, neurons in the lower chamber were fixed and stained in situ. Confocal images were acquired using identical microscope settings across groups, and 3 randomly selected fields per well were analyzed. For ATP/ADO measurements, conditioned medium was collected from the lower chamber and analyzed using commercial assay kits according to the manufacturers’ instructions.

### Cell Viability and Biocompatibility Assessment

Cell viability of PC12 cells was evaluated using a live/dead cytotoxicity kit (calcein-AM/PI, Invitrogen, USA) and a CCK-8 assay (Dojindo, Japan). For live/dead staining, cells were incubated with calcein-AM and PI working solution for 20–30 min at 37 °C and imaged by confocal microscopy (Leica, Germany). In each well, 3 randomly selected fields were acquired, and live/dead cells were counted with ImageJ; viability was calculated as live / (live + dead) × 100%. For CCK-8, reagent was added to each well and incubated for 1 h, and absorbance at 450 nm was measured with a Multiskan FC (Thermo, USA). Values were background-subtracted and normalized to control wells. Data are from at least three independent experiments.

### Intracellular ROS

Intracellular ROS levels were measured using a Total ROS Assay Kit (Beyotime Biotechnology, China) according to the manufacturer’s instructions. HT22 cells were seeded in 6-well plates at a density of 1 × 10^6^ cells/well and allowed to adhere overnight before treatments. After the indicated interventions, cells were incubated with the ROS probe diluted in serum-free medium for 15 min at room temperature in the dark. Cells were then washed with PBS, detached with trypsin without EDTA, and resuspended in PBS for flow cytometry (BD, USA). Mean fluorescence intensity (MFI) of the ROS probe was quantified using FlowJo software. Data are from at least three independent experiments.

### Mitochondrial membrane potential (MMP) measurement

MMP was evaluated using JC-1 (Beyotime Biotechnology, China). Cells were incubated with JC-1 staining solution, washed, and analyzed by flow cytometry, distinguishing red fluorescence (normal mitochondria) and green fluorescence (depolarized mitochondria).

Mitochondrial membrane potential was assessed using the JC-1 assay (Beyotime Biotechnology, China). HT22 cells were seeded in 6-well plates at a density of 1 × 106 cells/well and treated as indicated. At the end of treatments, cells were incubated with JC-1 working solution for 20 min at 37 °C in the dark. After incubation, cells were gently washed with JC-1 buffer, detached with trypsin without EDTA, and resuspended in JC-1 buffer for immediate analysis by flow cytometry (BD, USA). Red fluorescence (JC-1 aggregates, indicating polarized mitochondria) and green fluorescence (JC-1 monomers, indicating depolarized mitochondria) were recorded in appropriate channels. The mitochondrial membrane potential index was calculated as the ratio of red to green fluorescence (aggregate/monomer) using FlowJo software. Data are from at least three independent experiments.

### Intracellular ATP measurement

Intracellular ATP levels were quantified using an Enhanced ATP Assay Kit (Beyotime Biotechnology, China) according to the manufacturer’s protocol. At the end of the treatments, cells were lysed in ATP lysis buffer and cell lysates were transferred to black

96-well plates, mixed with ATP detection working solution. Luminescence was recorded using a Multiskan FC microplate reader (Thermo, USA). Data are from at least three independent experiments.

### Mitophagy assay

Mitophagy was evaluated using a commercial mitophagy detection kit (Dojindo, Japan) following the manufacturer’s instructions. After treatments, HT22 cells were incubated with the mitophagy probe and lysosome dye and MitoTracker (Dojindo, Japan). Samples were imaged using a laser scanning confocal microscope. For quantification, 3 random fields per coverslip were analyzed. Mitophagy was quantified by measuring mitochondria–lysosome colocalization using ImageJ. Data are from at least three independent experiments.

### Immunofluorescence staining

Cells grown on glass coverslips were washed with PBS and fixed in 4% paraformaldehyde for 30 min at room temperature. After washing, cells were permeabilized and blocked in 3% BSA containing 0.1% Triton X-100 for 30 min, followed by incubation with primary antibodies diluted in blocking buffer overnight at 4 °C. The next day, cells were washed and incubated with appropriate secondary antibodies for 1 h at room temperature in the dark, then counterstained with DAPI. Coverslips were mounted with antifade medium and imaged using a laser scanning confocal microscope under identical acquisition settings across groups.

For quantification, 3 randomly selected fields per coverslip were analyzed. The number of marker-positive cells or mean fluorescence intensity within defined regions of interest was measured using ImageJ. Values were averaged per coverslip and considered one biological replicate; data represent mean ± SEM from 3 independent experiments.

The dilution ratios of primary antibodies were listed below, Mouse anti-P2Y12R(1:300; AnaSpec.AS-55043A).Goat anti- Iba-1 (1:500; Abcam, ab18207).Rabbit anti-iNOS(1:500; Abcam, ab210823), Rabbit anti-TUJ1(1: 500; Abcam, ab18207), Chicken anti-GFAP(1:5000; Abcam,ab4674), Mouse anti-NF200 (1:500; sigma, N5389), Rabbit anti-MBP(1:500; Abcam, ab218011), Rabbit anti-LC3(1: 500; CST, #12741), Rat anti-Neun (1:1000; Abcam, ab279297), Mouse anti-Tomm20 (1:500; Abcam, ab56783).

### RT-PCR

Total RNA was extracted using an RNA isolation kit (Omega, China) according to the manufacturer’s protocol. Equal amounts of RNA 1 µg were reverse transcribed into cDNA using a commercial reverse transcription kit (Omega, China). Quantitative RT-PCR was performed on a LightCycler 480 system (Takara,Japan) using SYBR Green chemistry. Each sample was run in technical triplicates. Relative gene expression was calculated using the 2^-ΔΔCt method, with ACTIN as the internal reference gene. For each condition, n represents independent biological samples, and mean Ct values of technical triplicates were used for analysis. Primer sequences are listed in Table S1.

### Western Blot

Cells or tissues were lysed in RIPA buffer, and protein concentrations were determined by BCA assay(Beyotime Biotechnology, China). Equal amounts of protein were denatured, separated by SDS-PAGE, and transferred to PVDF membranes. Membranes were blocked with 5% non-fat milk and incubated with primary antibodies overnight at 4 °C, followed by secondary antibodies. Bands were visualized with ECL reagents (Thermo Fisher, USA) and imaged on a chemiluminescence system. Densitometry was performed using ImageJ,

with GAPDH as the loading control. The dilution ratios of primary antibodies were listed below, and the GAPDH (1:4000; Abcam, ab8245,37KD) was included inside as the loading control, rabbit anti-Pink1(1:1000; CST, #85325,50KD), mouse anti-Parkin (1:1000; CST, #4211,50KD), rabbit anti-LC3(1:1000; CST, #12741,14 KD,16KD).

### Ex vivo study

The protocol we applied is a modification of the interface method described by Chen et al [29]. Neonatal C57BL/6 mice (postnatal day 3–5, both sexes) were obtained from the Guangdong Animal Experiment Center and used immediately for experiments after arrival. All animal experimental procedures were approved by Institutional Animal Care and Use Committee, Sun Yat-Sen University (SYSU-IACUC-2023-001598). Mice were euthanized by rapid decapitation under deep isoflurane anesthesia, and the spinal cords were quickly removed under a stereomicroscope. Tissues were then cut into 200 μm cross sections with a McIlwain chopper (Ted Pella, USA) for ex vivo spinal slice culture (organotypic slice culture). The spinal slices were placed on the 0.4 μm semi-porous membrane (Millipore) pre-loaded with CMC hydrogel and the lower chamber was filled with culture medium (50% mem, 25% horse serum, 25%HBSS, 6.5 mg/ml D-glucose). 200 μm H_2_O_2_ was used to simulate oxidative stress during SCI. Tunel assay and immunohistochemistry were performed after the 3D organotypic slice culture.

### Animal surgery

Eight-week-old female C57BL/6 mice were purchased from the Guangdong Animal Experiment Center and housed under specific pathogen-free conditions (22 °C–24 °C, 12 h light/dark cycle) with free access to food and water. All animal procedures were approved by the Institutional Animal Care and Use Committee of Sun Yat-Sen University (SYSU-IACUC-2023-001598). Mice were anesthetized with 2.5–3% isoflurane, and a midline dorsal incision was made to expose the T10 vertebra. A 5 mm-wide, 0.1 mm-thick microforceps (11295-00, F.S.T.) was used to produce a contusion injury by compressing the exposed spinal cord circumferentially for 5 s. After injury, muscle layers were sutured and the skin was closed. Mice were kept on a warming pad until fully recovered from anesthesia, and bladders were manually expressed daily until spontaneous urination returned. Animals were monitored daily for general condition and wound healing. Mice whose BMS hindlimb locomotor score was greater than 0 on postoperative day 1 (indicating unsuccessful injury) were excluded from subsequent analyses.

### Nanoparticles Treatment for SCI Mice

Mice were randomly assigned to four groups: Spinal Cord Injury (SCI), ROS-responsive nanoparticle treatment (TK-Nano), P2Y-Nano treatment (P2Y-Nano), and combined ROS-responsive P2Y12-delivery treatment (P2Y-TK-Nano). Group allocation was performed by an investigator not involved in subsequent injections or outcome assessments. All animals underwent the same complete SCI procedure as described above. From day 0 (2 h after SCI) to day 6 post-injury, mice in the SCI group received 50 µl sterile saline via tail vein injection, whereas mice in the TK-Nano, P2Y-Nano, and P2Y-TK-Nano groups received 50 µl of their respective nanoparticle formulations (10 mg ml⁻¹) by tail vein injection on the same schedule. Experimental endpoints were set at 1 week to evaluate acute-phase effects, at 4 weeks to assess subacute-phase outcomes, and at 8 weeks to examine chronic-phase structural and functional recovery.

### Behavioral assessment

The Basso Mouse Scale (BMS) was used to assess hindlimb locomotor recovery on postoperative day 1 and then at weekly intervals for a total of 8 weeks, with manual bladder evacuation performed before each assessment. Mice were placed individually in an open field and allowed to move freely for 4 min while being observed. Each hindlimb was scored separately according to the standard BMS criteria, and the mean of the left and right hindlimb scores was taken as the BMS score for that mouse at each time point. Two trained observers, blinded to group allocation and treatment, scored all animals independently; their scores were then averaged for statistical analysis. For behavioral outcomes, n refers to the number of mice per group.

### Electrophysiological assessment

Motor Evoked Potentials (MEP) were measured at four weeks post-surgery to assess motor function recovery. Mice were anesthetized, and a craniotomy was performed to access the motor cortex. Stimulation electrodes were placed in the motor cortex, and the sciatic nerve was exposed for signal recording. Experimental parameters included a stimulation frequency of 10 Hz and voltage of 5 V, with subsequent recording of potential waveforms for amplitude and latency analysis.

### Histological analysis

At Experimental endpoints, spinal cord tissues were collected after deep anesthesia and thoracotomy. A syringe was used to perfuse 50 mL of cold saline through the left ventricle to clear blood. Approximately 0.5 cm of damaged spinal tissue was preserved at –80 °C for Western blotting and RT-PCR. Remaining tissues were perfused with 50 mL of 4% paraformaldehyde and stored at 4 °C for sectioning.

### Electron microscopy

To examine mitochondrial ultrastructure in neurons, mice were deeply anesthetized and perfused with saline. A 3 mm spinal cord segment centered on the lesion site was dissected and trimmed into 2 × 2 mm tissue blocks, which were fixed in 2.5% glutaraldehyde at room temperature for 2 h and then stored at 4 °C until further processing.

Neuronal profiles in spinal cord sections were identified based on characteristic ultrastructural features, including the presence of multivesicular bodies and vesicle-rich presynaptic terminals within the same field. Mitochondria within these neuronal regions were classified as normal or damaged according to cristae integrity and matrix density.

### Statistics

Statistical analyses were performed using SPSS 20.0 software. Data are expressed as means ± standard deviations (SDs). Before hypothesis testing, data distributions were examined for normality (Shapiro–Wilk test) and homogeneity of variances (Levene’s test). Group comparisons were performed using unpaired t-tests (Mann-Whitney) or one-way analysis of variance (ANOVA), with a significance threshold of *P* < 0.05. The sample size was determined with reference to our previous work and published SCI studies. For the in vivo SCI experiments, the sample size was set at *n* = 6 mice per group. For ex vivo and in vitro experiments, each experimental condition was performed in three independent biological replicates (*n* = 3), with technical duplicates or triplicates when appropriate.

## Supplementary information

Original WB Data

Supplementary Materials

## Data Availability

All data are available in the main text or the supplementary materials.
