# Supplementary material for: Dual-targeting nanoparticles enhance microglial P2Y12R expression to promote neuronal mitophagy for repairing spinal cord injury
Source: Cell Death Dis. 2026 Apr 19;17(1):516. doi: 10.1038/s41419-026-08596-2 (PMC13221473; doi:10.1038/s41419-026-08596-2)

**Fig.2B**

P2Y12R


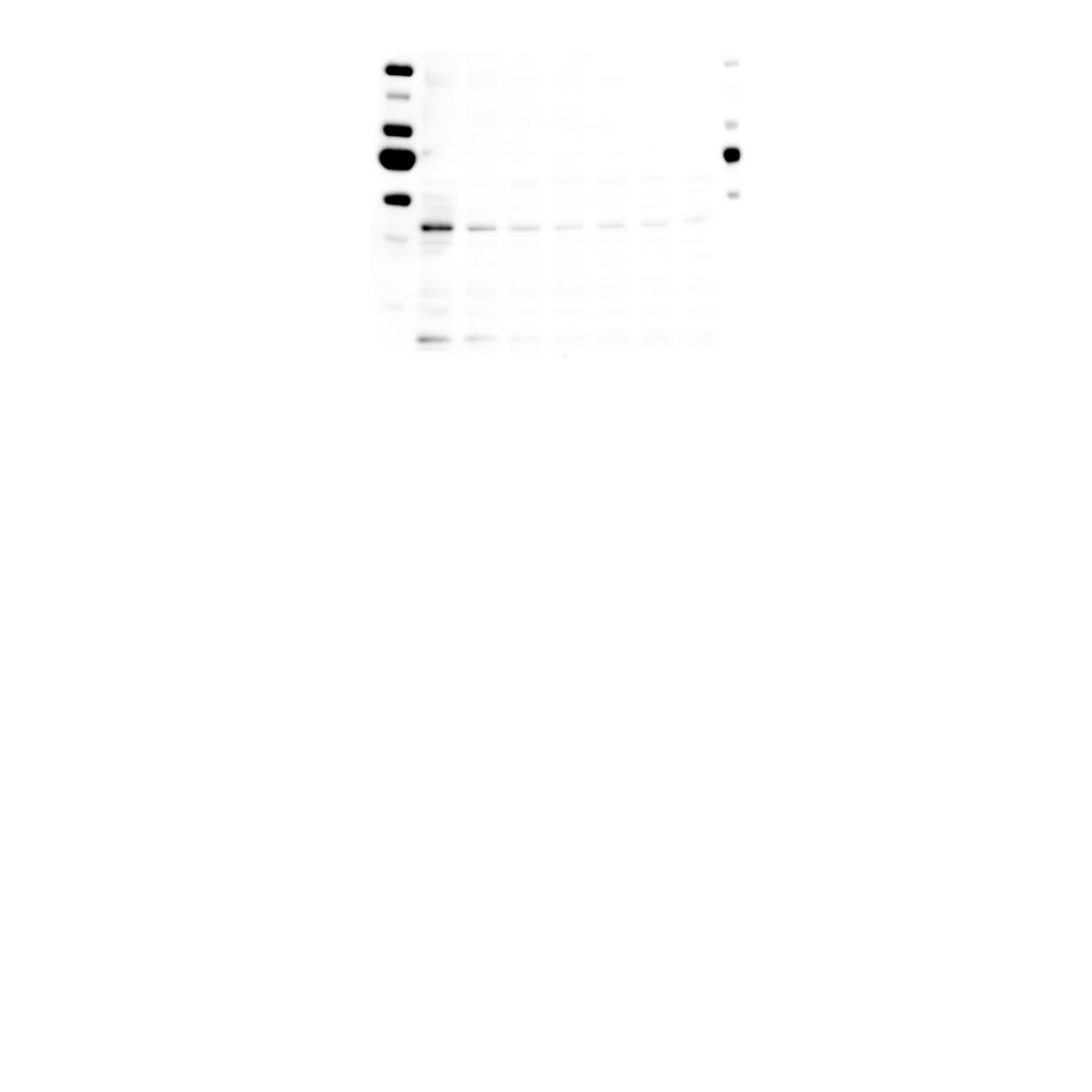


GAPDH


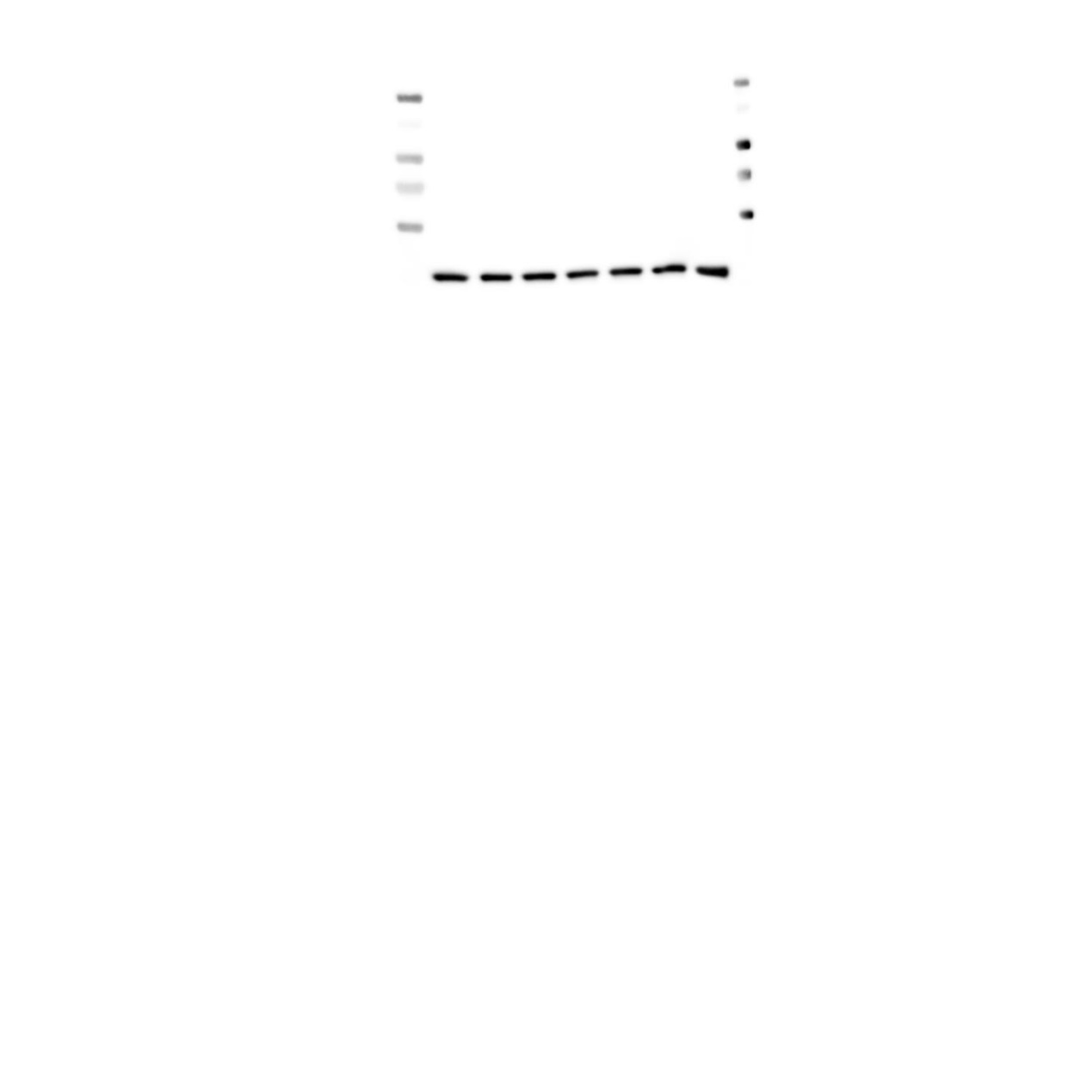


**Fig.3H**

Pink1





Parkin





LC3


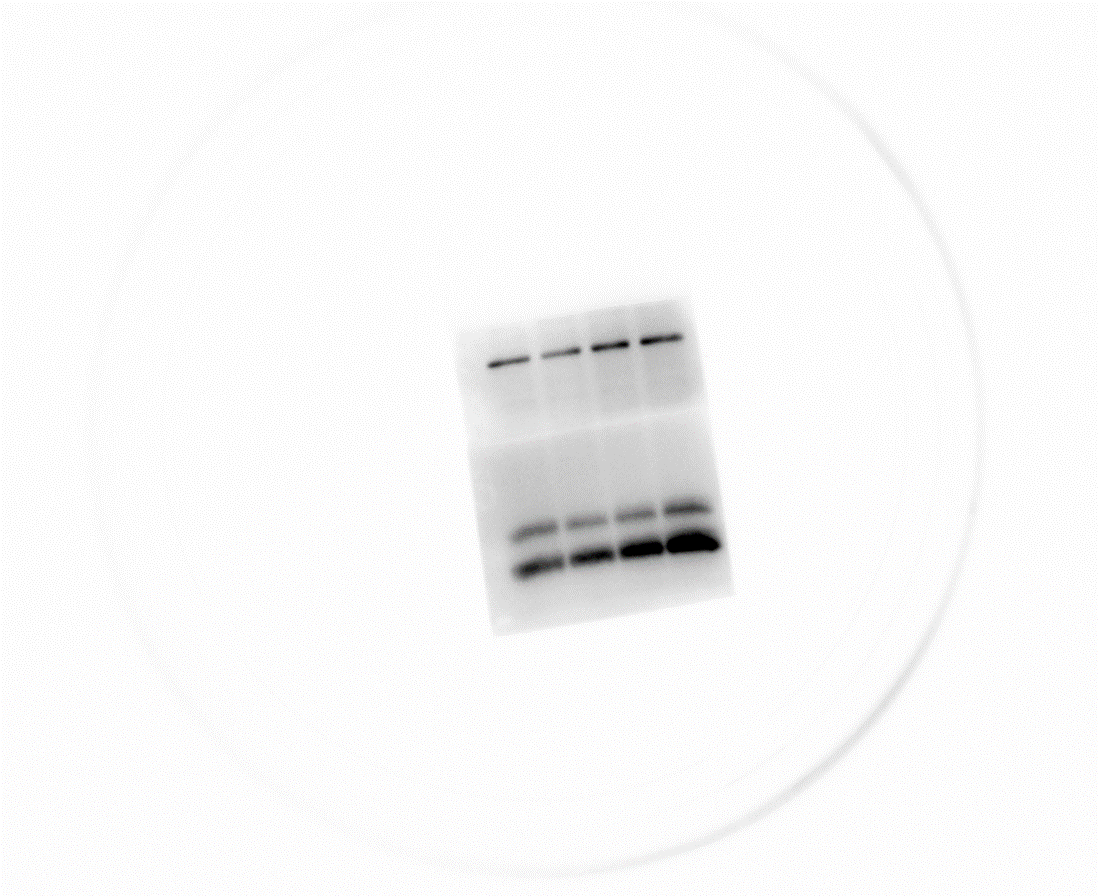


GAPDH





**Fig.4H**

Pink1





Parkin





LC3





GAPDH





**SI-5E**

Pink1


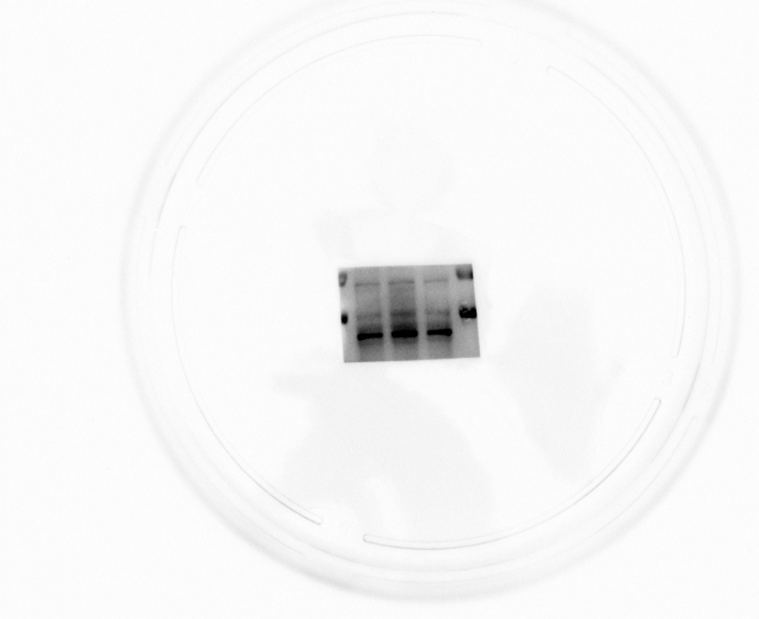


Parkin


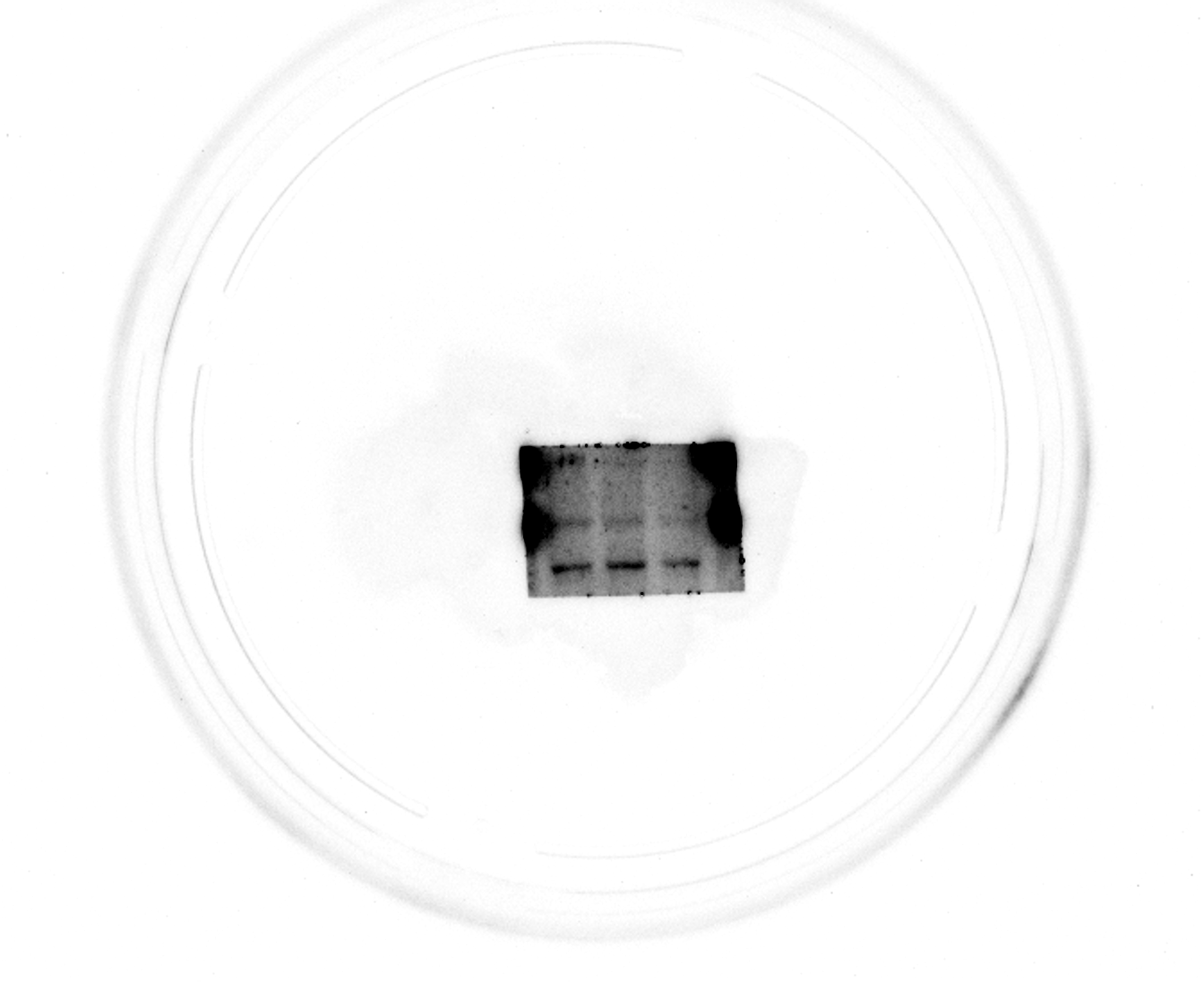


LC3&GAPDH


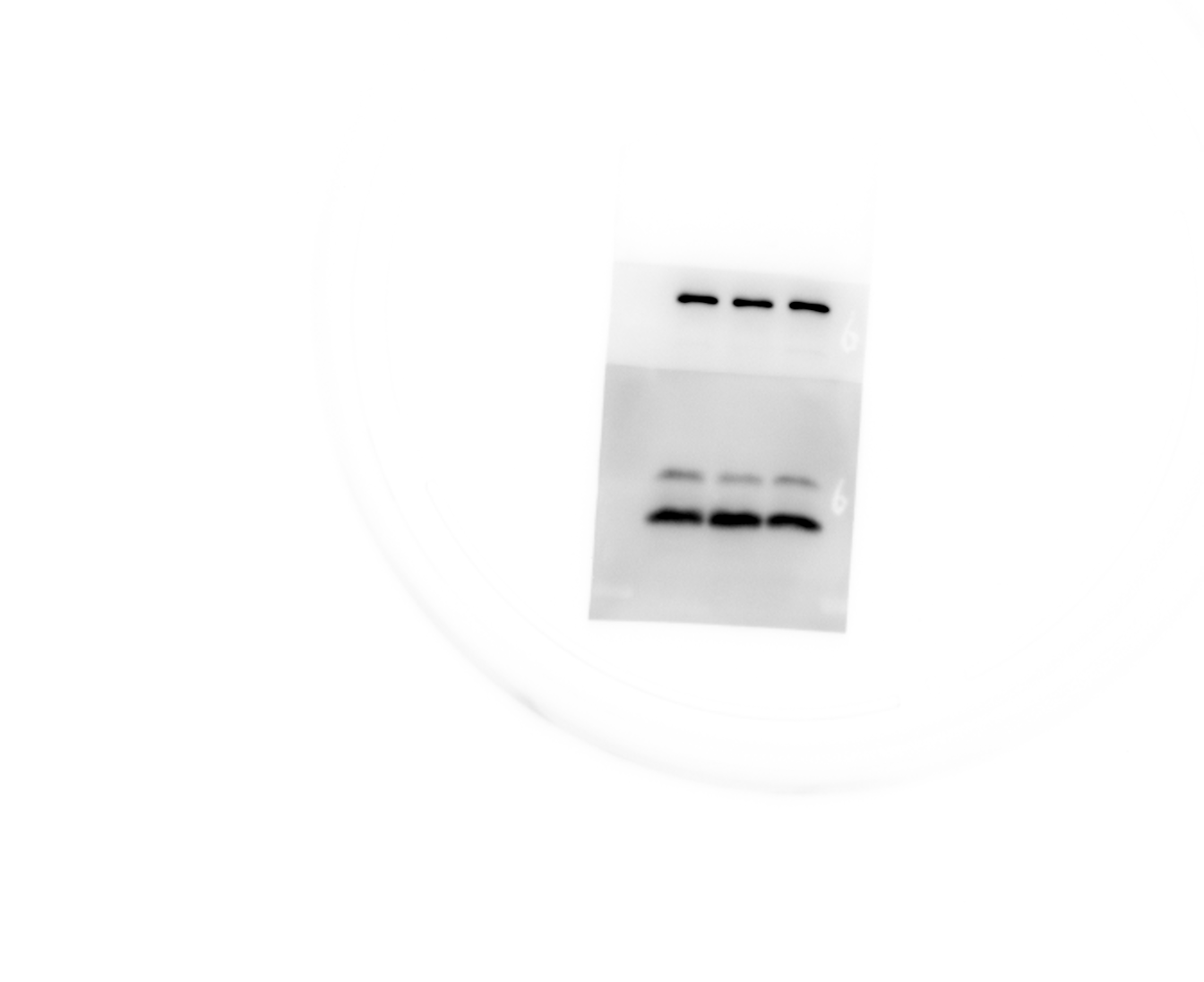


**SI-6C**

Pink1


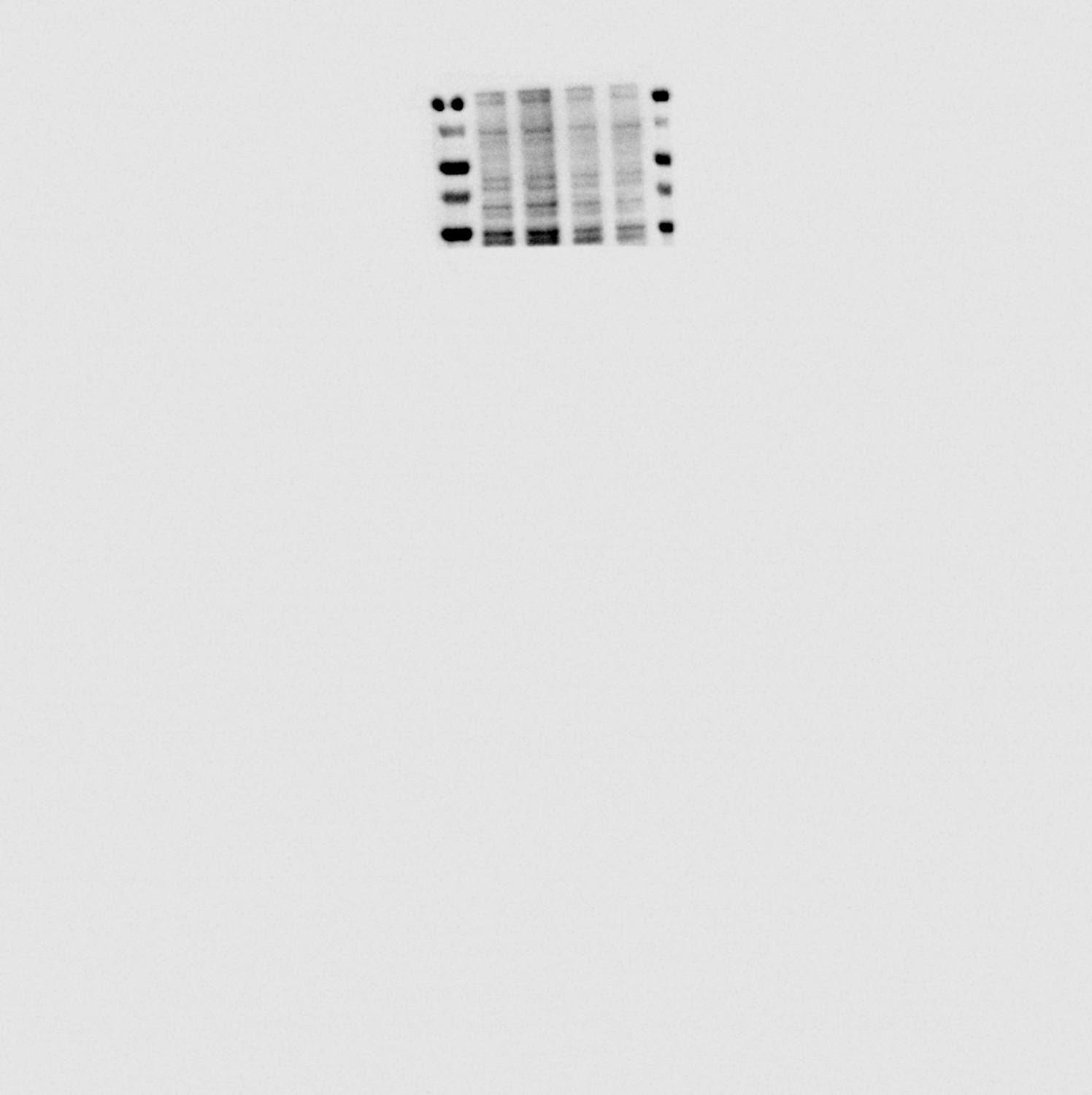


Parkin


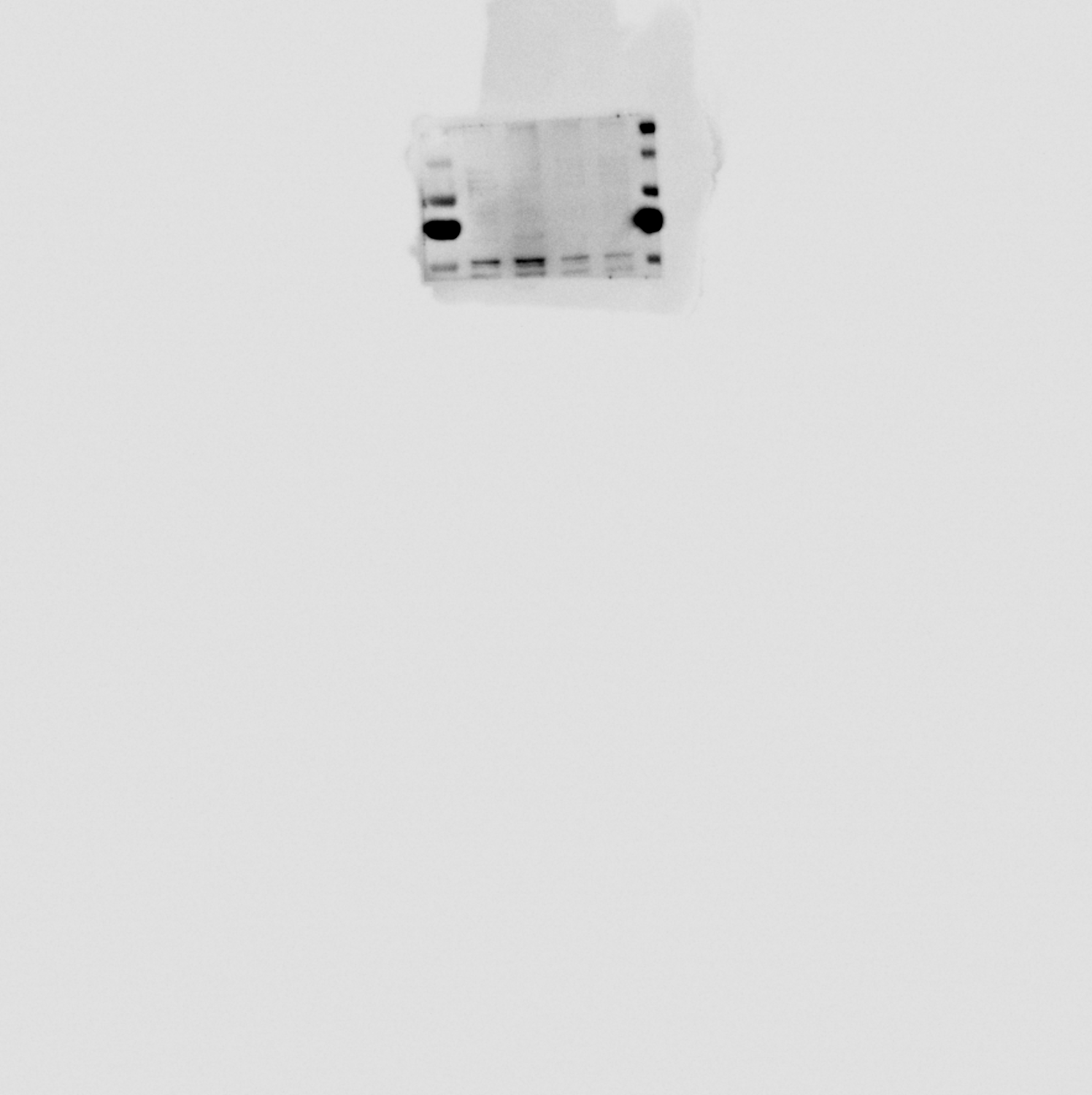


LC3


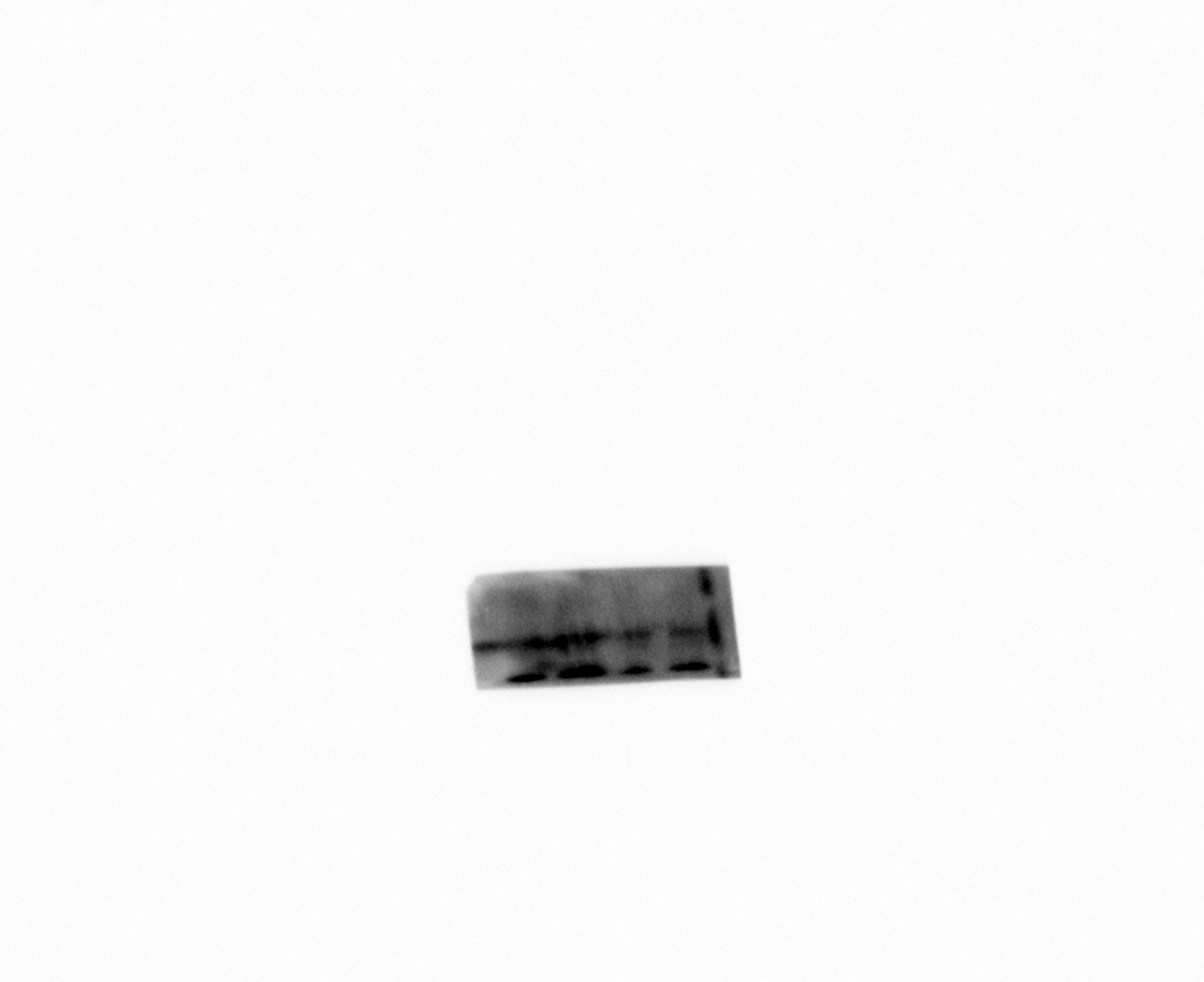


GAPDH


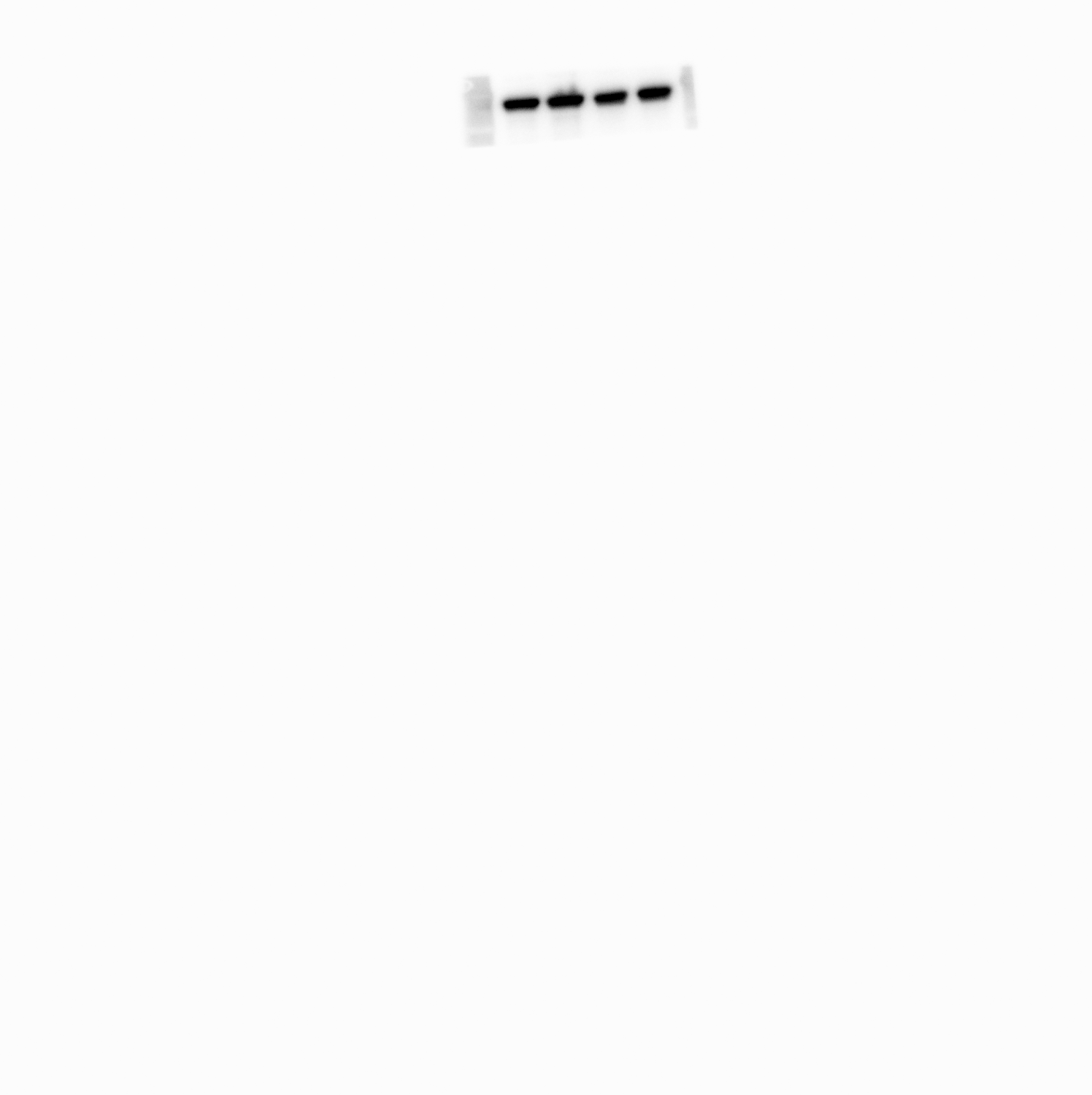


**SI-6D**

Pink1


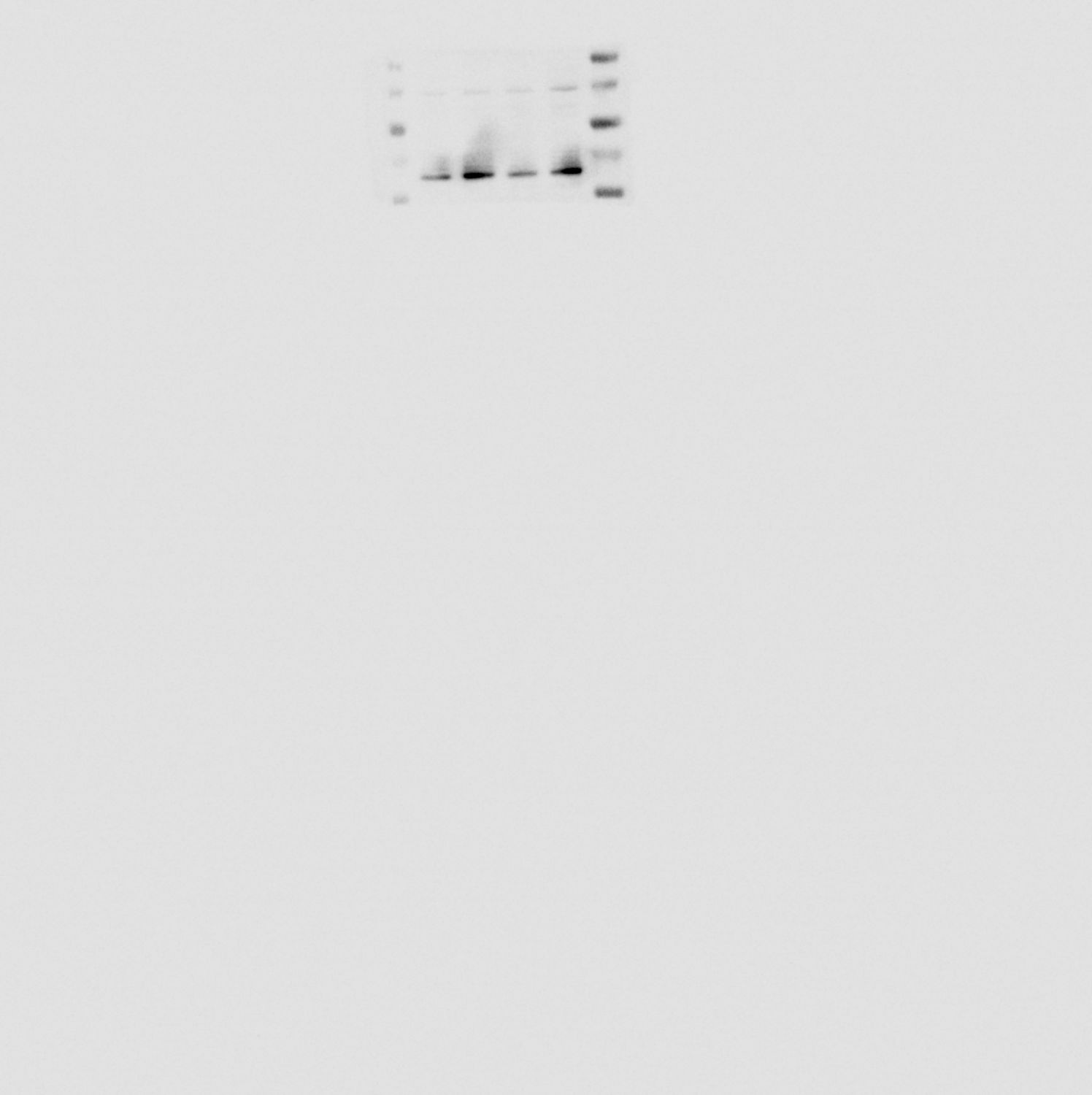


Parkin


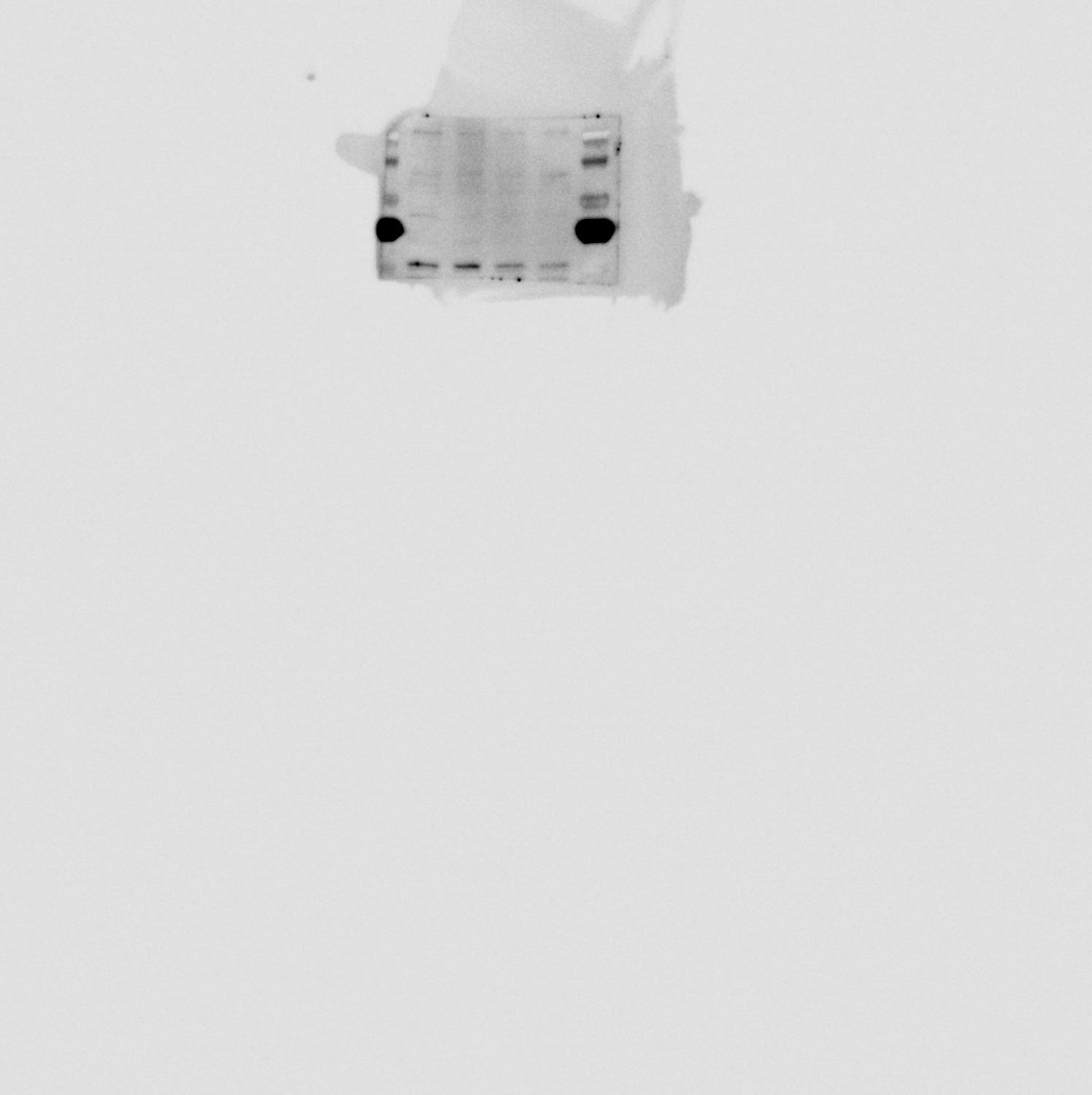


LC3


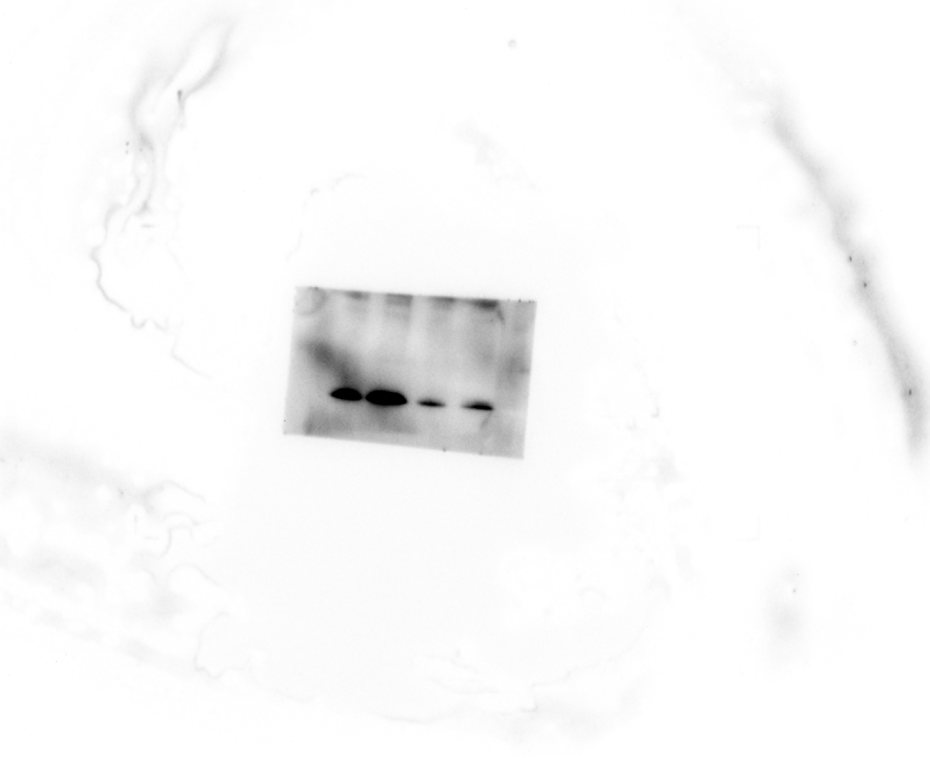


GAPDH


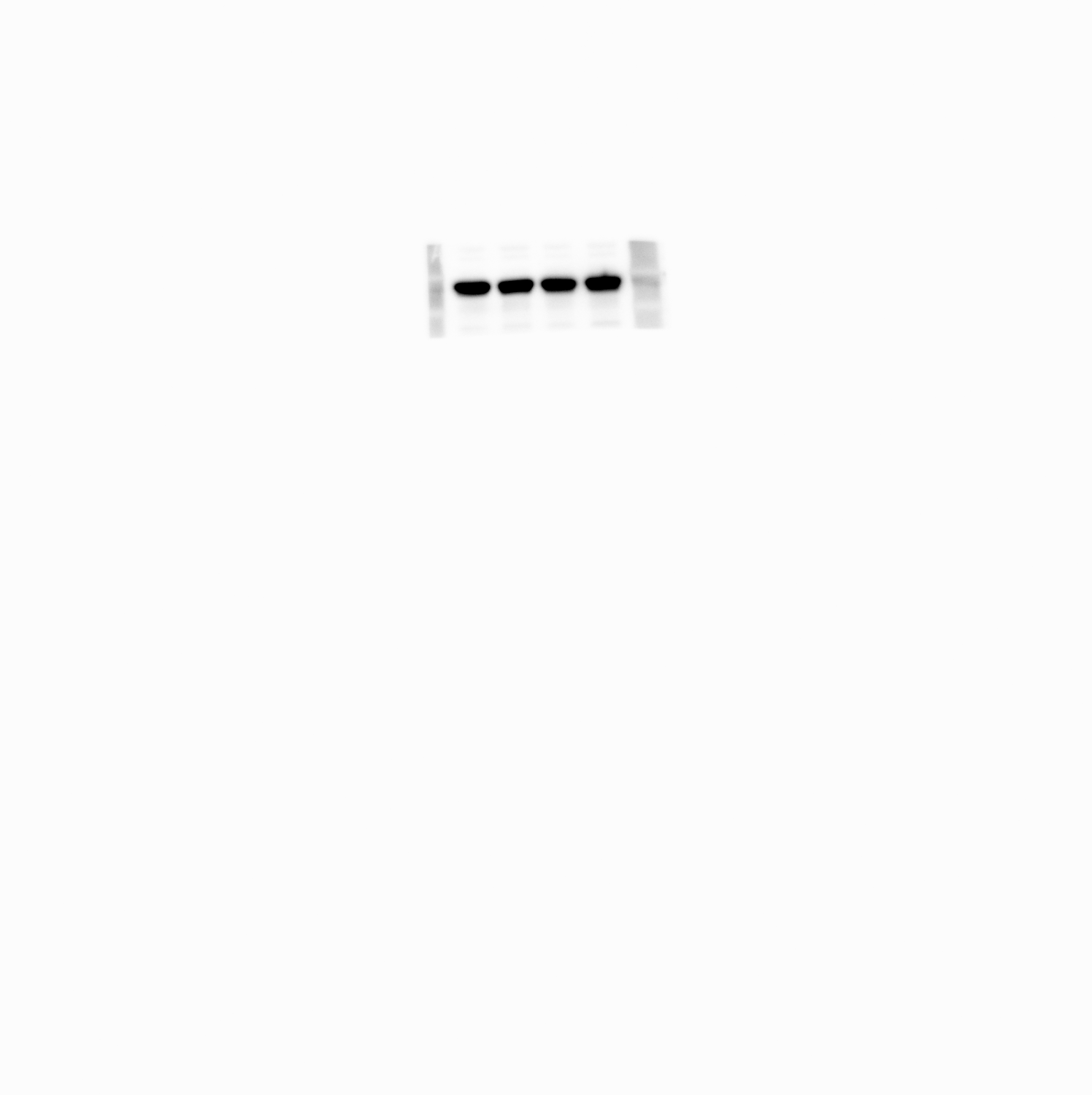


**SI-7H**

Pink1


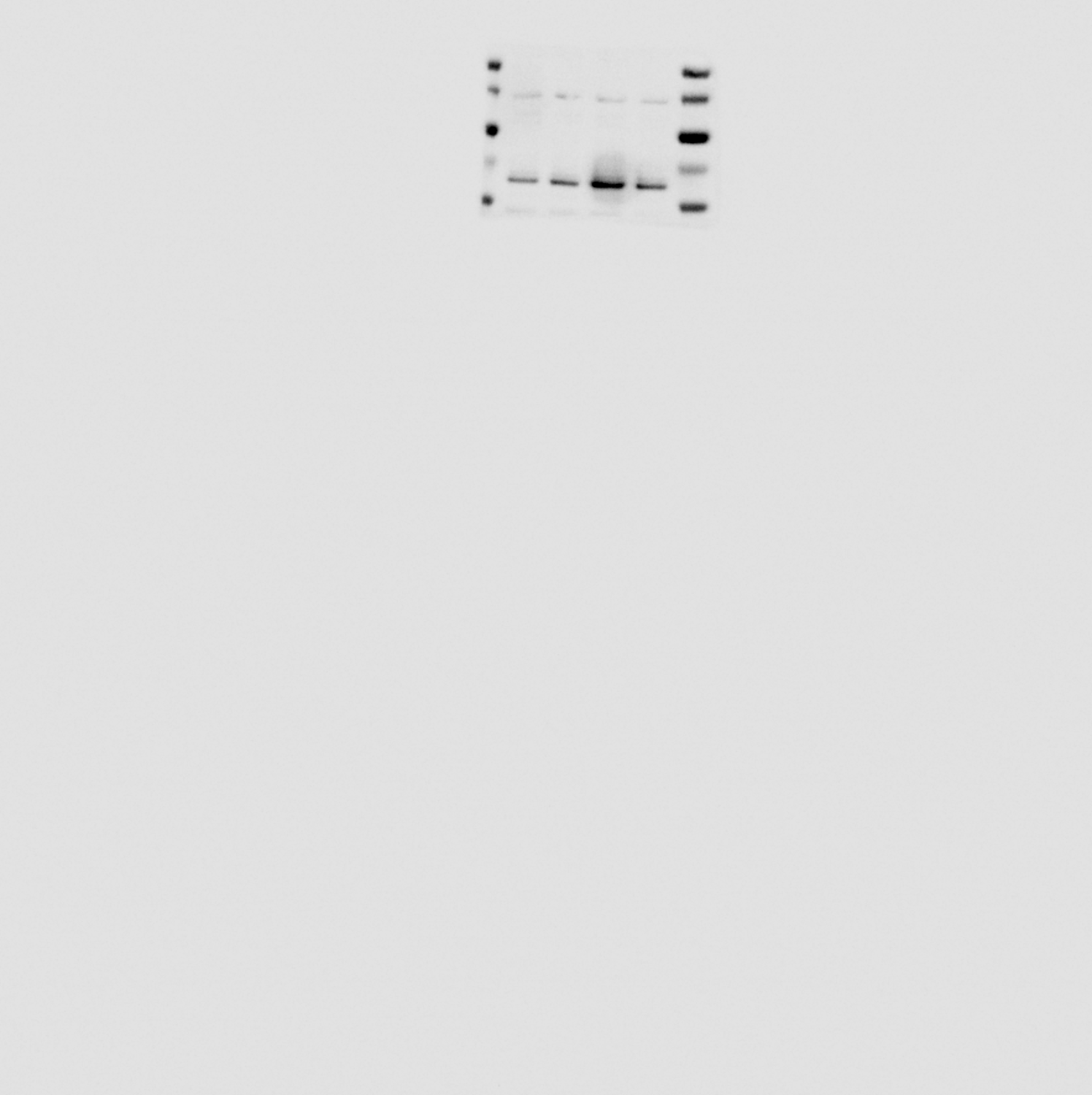


Parkin


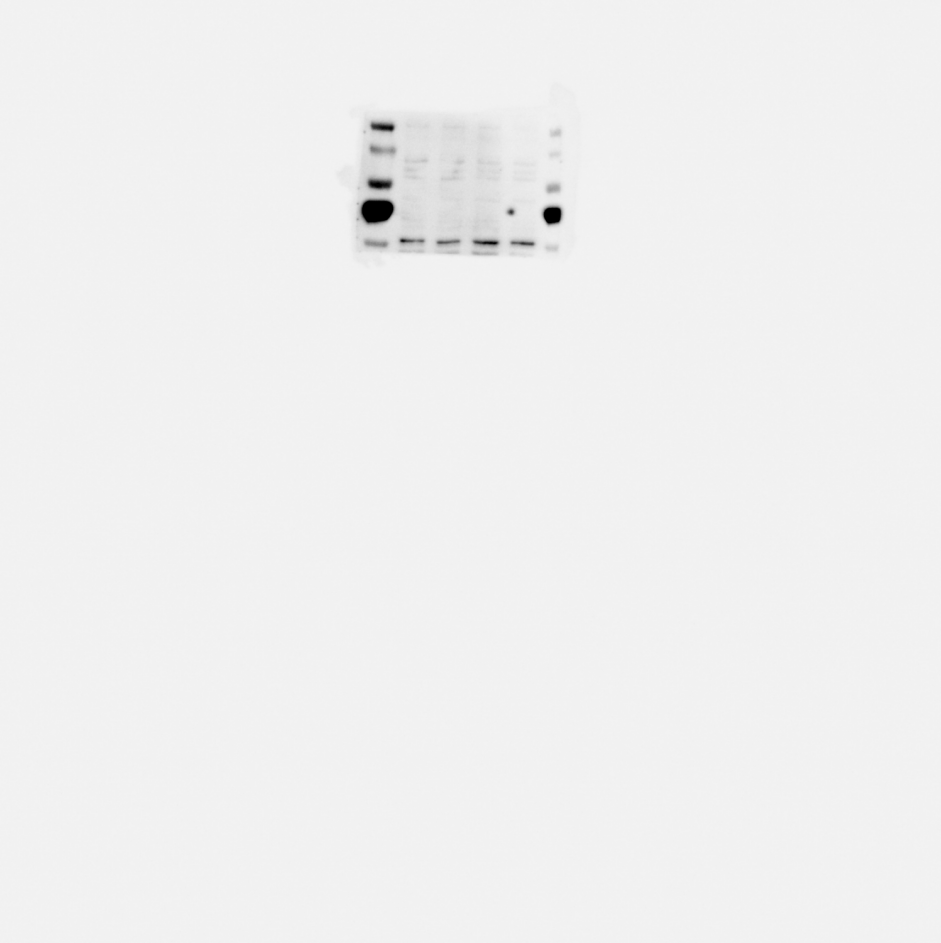


LC3


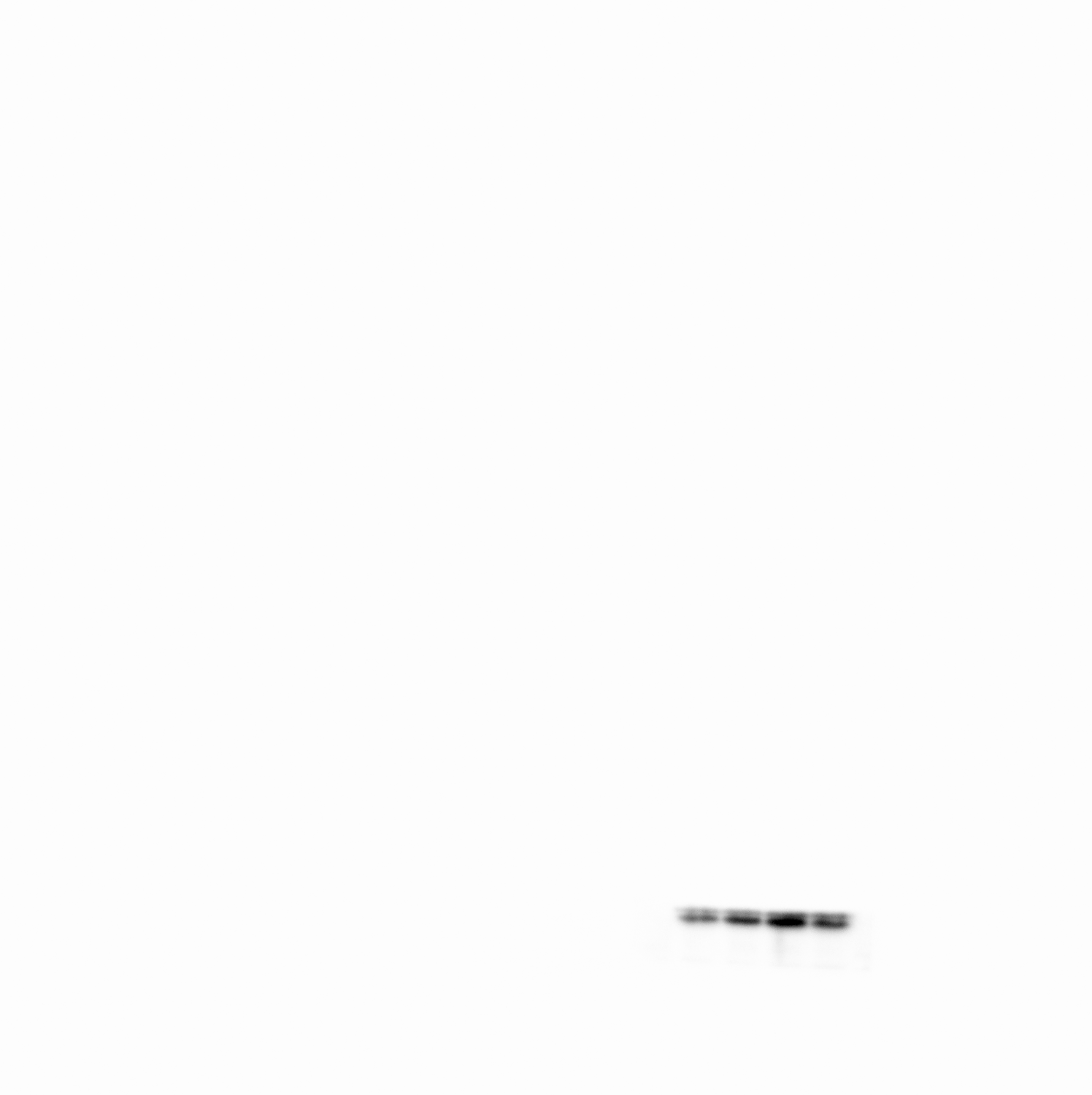


GAPDH


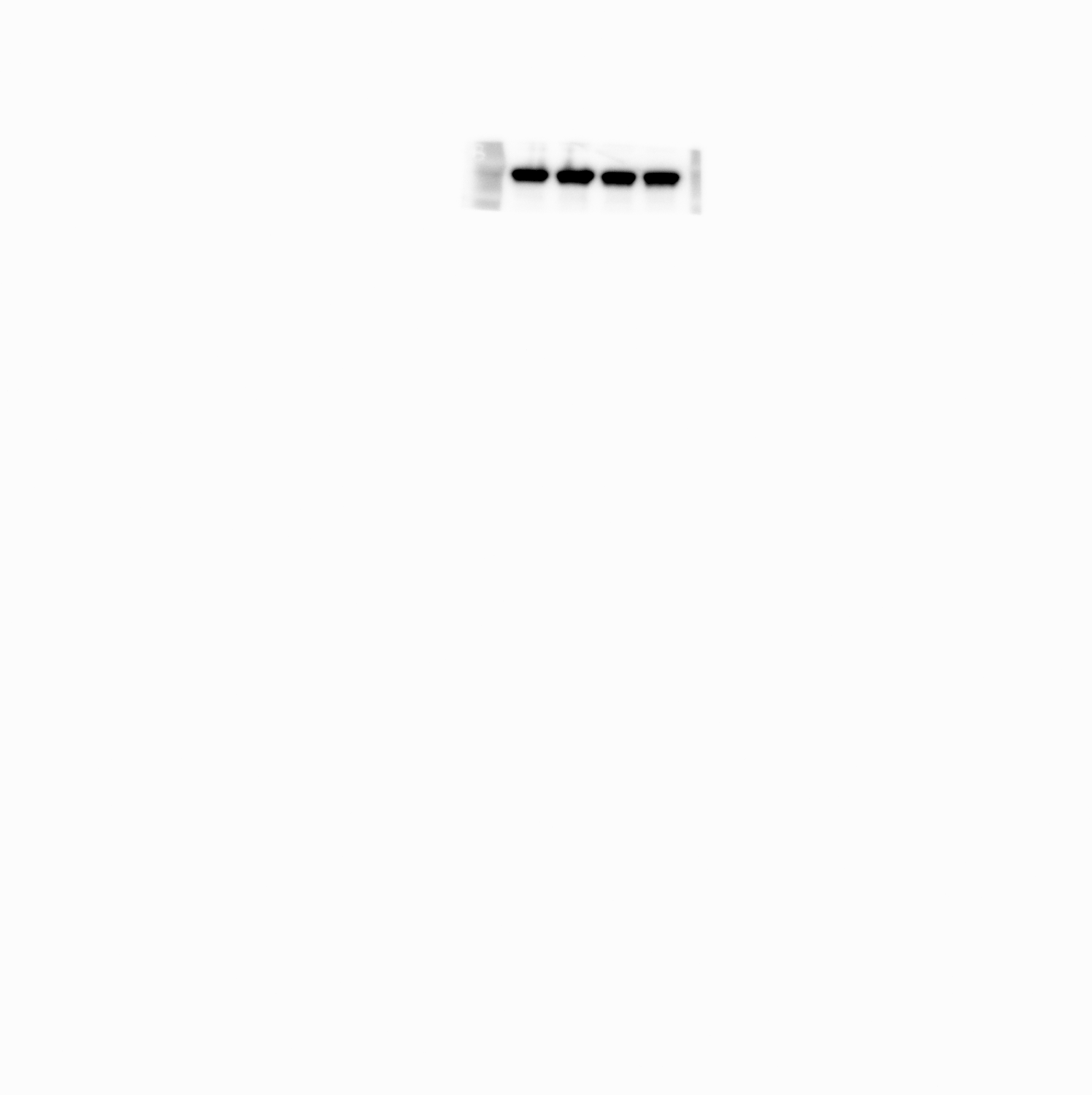

Supplement: Supplementary file 1 — Original WB Data [file 41419_2026_8596_MOESM1_ESM.docx]
