# Supplementary material for: Dual-targeting nanoparticles enhance microglial P2Y12R expression to promote neuronal mitophagy for repairing spinal cord injury
Source: Cell Death Dis. 2026 Apr 19;17(1):516. doi: 10.1038/s41419-026-08596-2 (PMC13221473; doi:10.1038/s41419-026-08596-2)
Supplement: Supplementary file 2 — Supplementary Materials [file 41419_2026_8596_MOESM2_ESM.docx]

Supplementary Materials for

Dual-Targeting Nanoparticles Enhance Microglial P2Y12R Expression to Promote Neuronal Mitophagy for Repairing Spinal Cord Injury

Zhenming Tian^123†^, Hong Li ^123†^, Yunheng Jiang^123†^, Huiye Wei^4^, Yubao Lu^123^, Senyu yao^123^, Mao Pang^123^**^*^**, Xintao Shuai^4^**^*^**, Bin Liu^123^ **^*^**, Limin Rong ^123^**^*^**

Correspondence to: ronglm@mail.sysu.edu.cn

**This PDF file includes:**

Figures. S1 to S11

Tables S1-S2

**Figure. S1.**


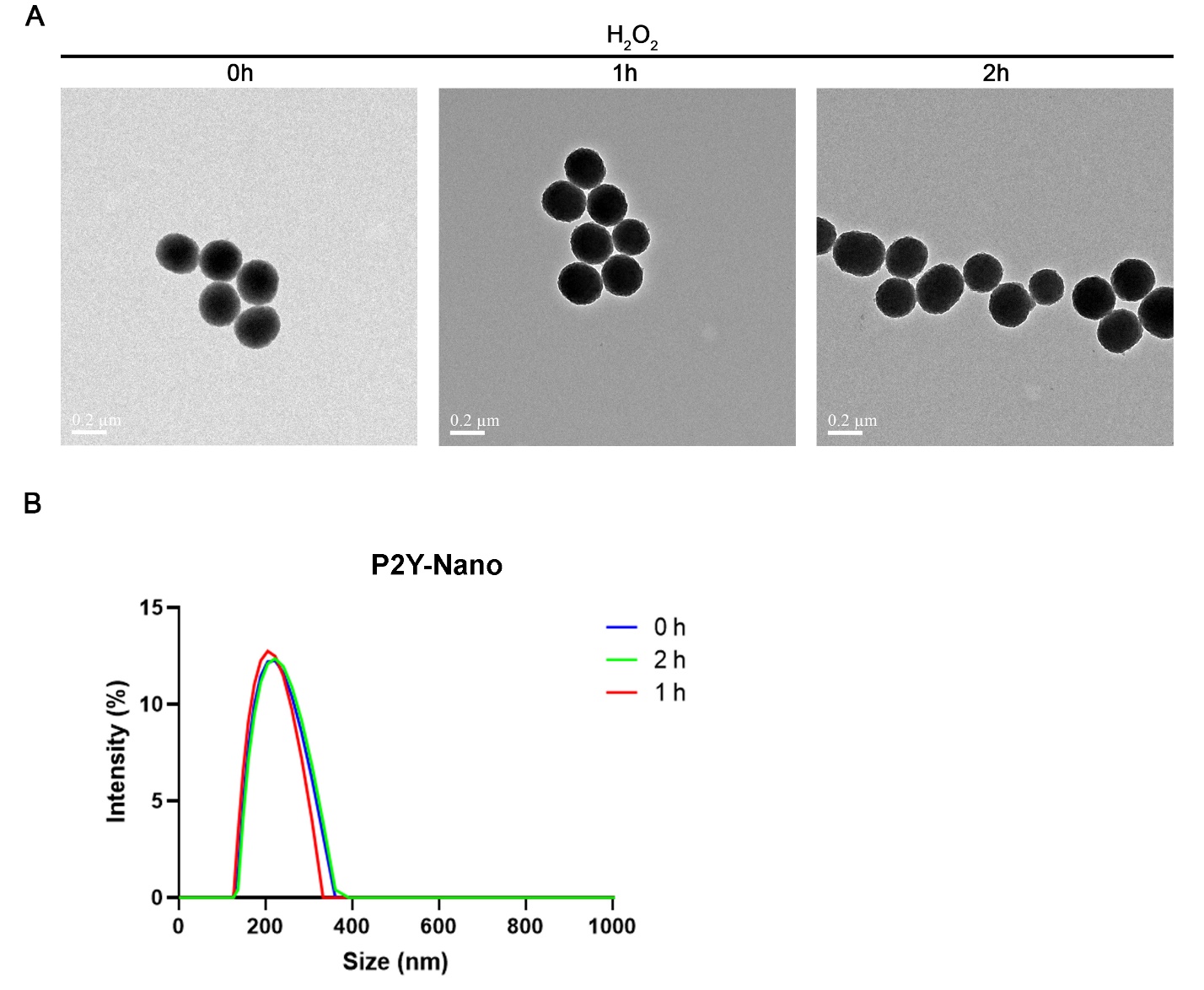


**ROS-Responsive Properties of P2Y-Nano**

(A) TEM images depicting the ultrastructural morphology of P2Y-TK-Nano; (B) Particle size analysis of P2Y-Nano in ROS-rich environments

**Figure. S2.**


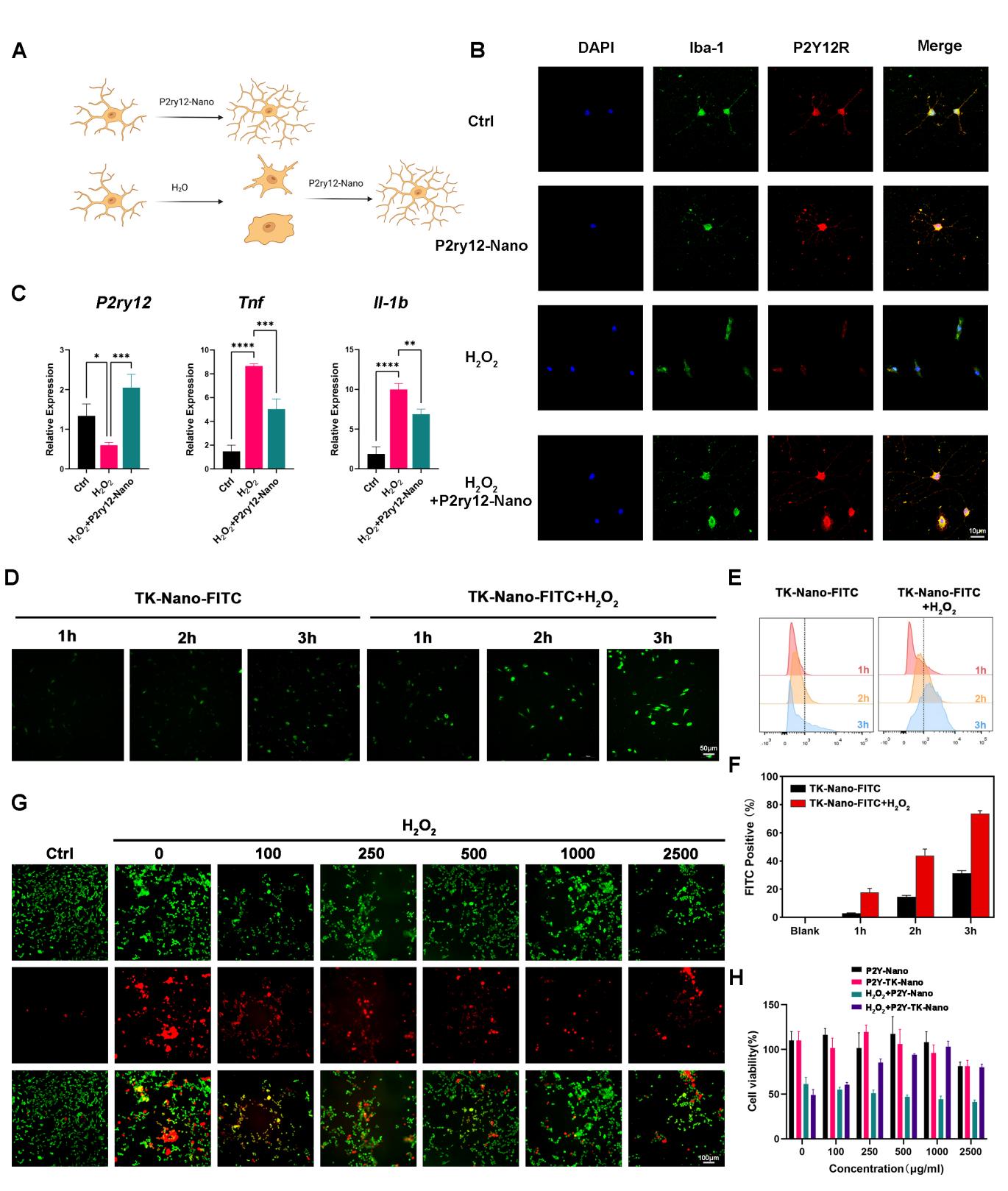


**Cellular Uptake, ROS-Responsive Behavior, and Cytocompatibility Analysis of P2Y-TK-Nano.**

(A) Schematic of P2ry12-Nano transfection in H₂O₂-activated microglia; (B) Immunofluorescence imaging of restored P2Y12R expression post-transfection; (C) Quantification of P2ry12 and pro-inflammatory cytokines in transfected microglia(samples, n=3）; (D) FITC fluorescence detection in primary microglia co-incubated with TK-Nano-FITC; (E) Flow cytometry analysis of panel D conditions; (F) Quantitative analysis of panel E(samples, n=3）; (G) Live/dead-stained viability of HT22 cells exposed to P2Y-Nano under ROS-rich conditions; (H) CCK-8 assay profiling HT22 viability post-P2Y-Nano treatment in normal/ROS-rich environments (samples, n=3). Data were presented as mean ± SD. Results were analyzed by One-way ANOVA. Significance: *P < 0.05, **P < 0.01, ***P < 0.001，****P < 0.0001.

Figure. S3.


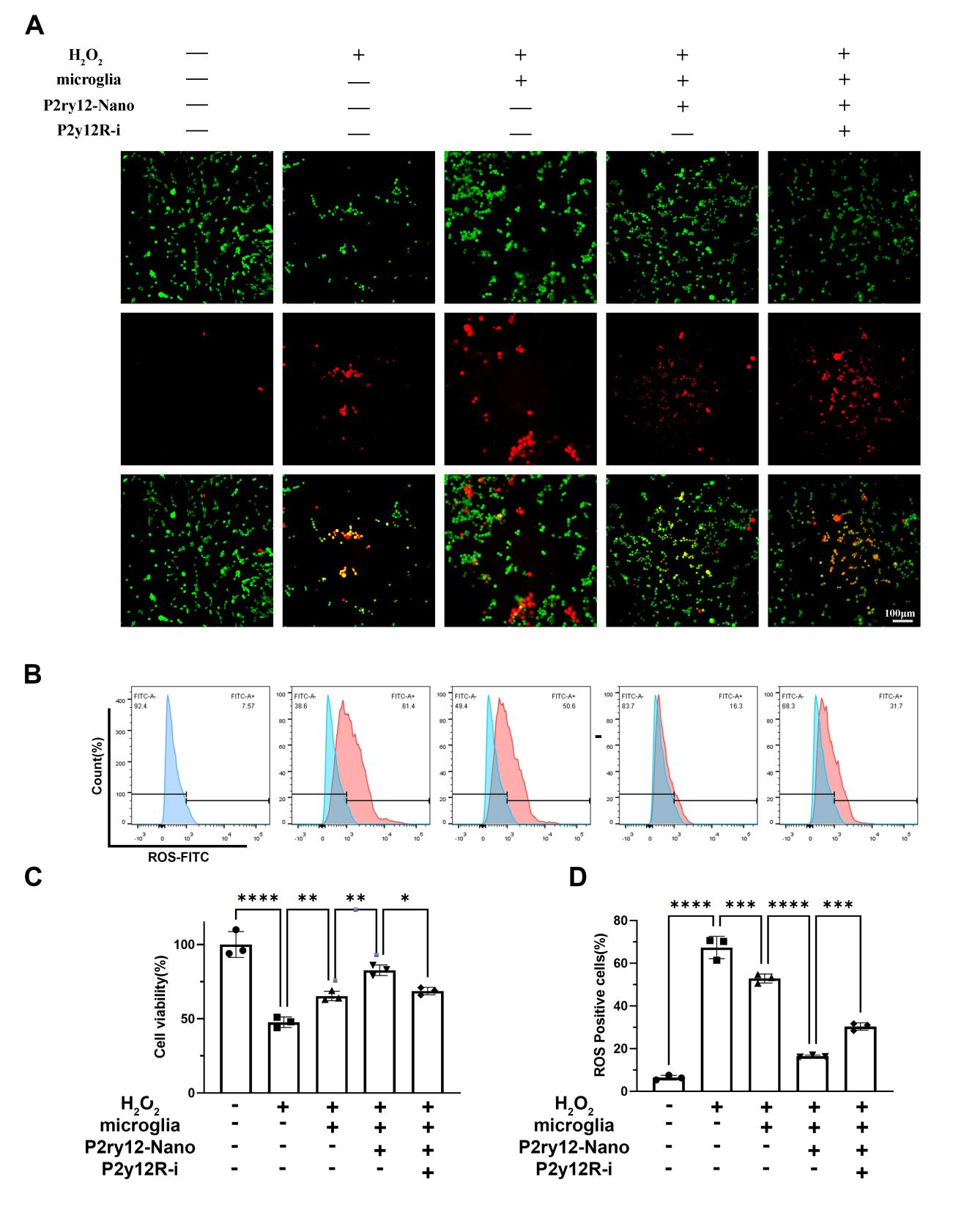


**Protective effects of P2Y12R overexpressed microglia on neurons under oxidative stress.**

(A) Live/dead staining of HT22 cells treated by P2Y12R-overexpressing microglial and P2Y12R inhibitor in ROS-rich environments; (B) Flow cytometry analysis of ROS levels in HT22 cells co-cultured with P2Y12R-overexpressing microglia; (C) Quantification of neuronal viability in (A) (samples, n=3); (D) Quantitative analysis of neuronal ROS levels in (B) (samples, n=3). Data were presented as mean ± SD. Results were analyzed by One-way ANOVA. Significance: *P < 0.05, **P < 0.01, ***P < 0.001，****P < 0.0001.

Figure. S4.


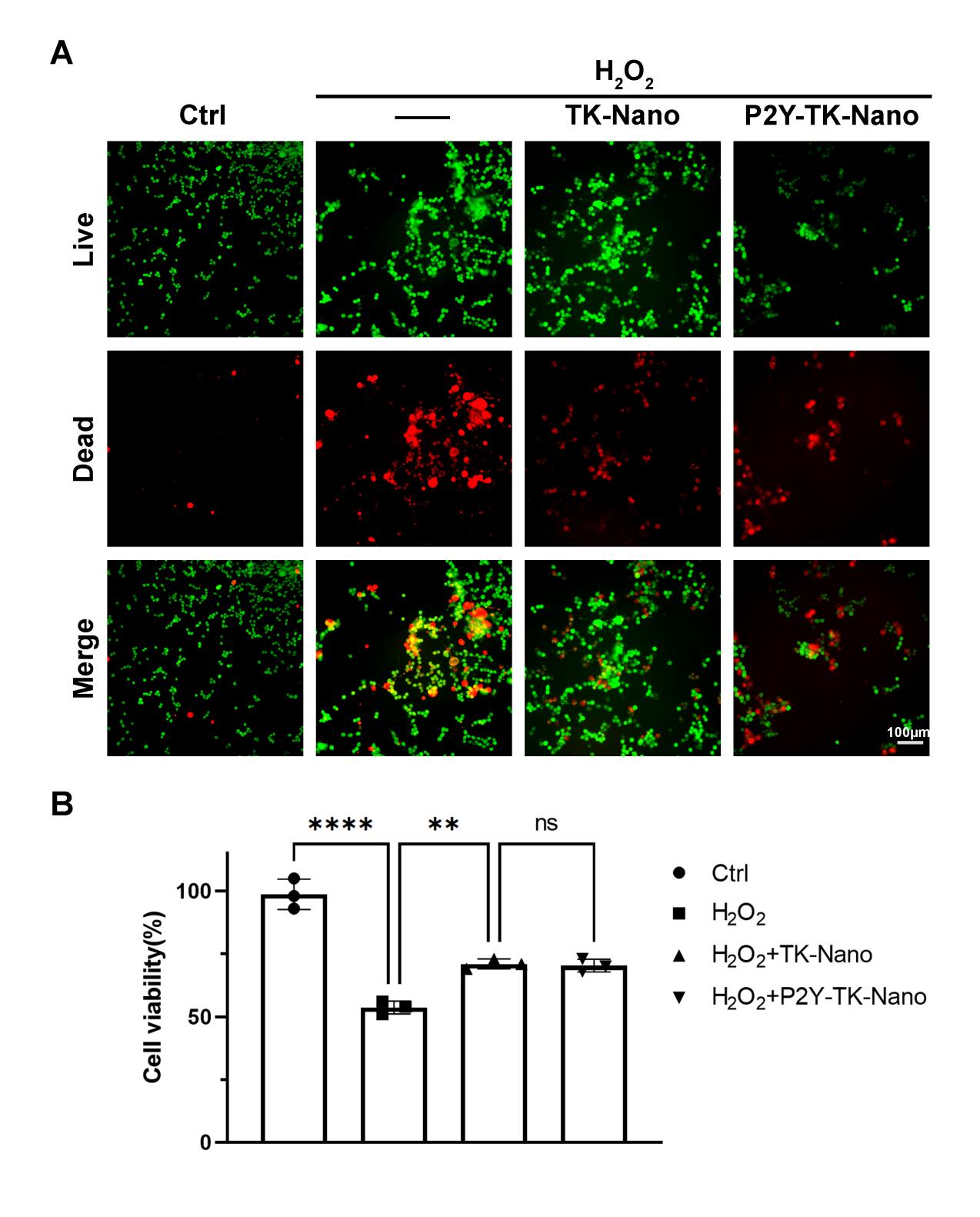


**Protective effects of ROS-responsive on neurons under oxidative stress.**

(A) Live/dead staining of HT22 cells treated by P2Y-Nano and TK-Nano in ROS-rich environments; (B) Quantification of neuronal viability in (A) (samples, n=3). Data were presented as mean ± SD. Results were analyzed by One-way ANOVA. Significance: *P < 0.05, **P < 0.01, ***P < 0.001，****P < 0.0001.

Figure. S5


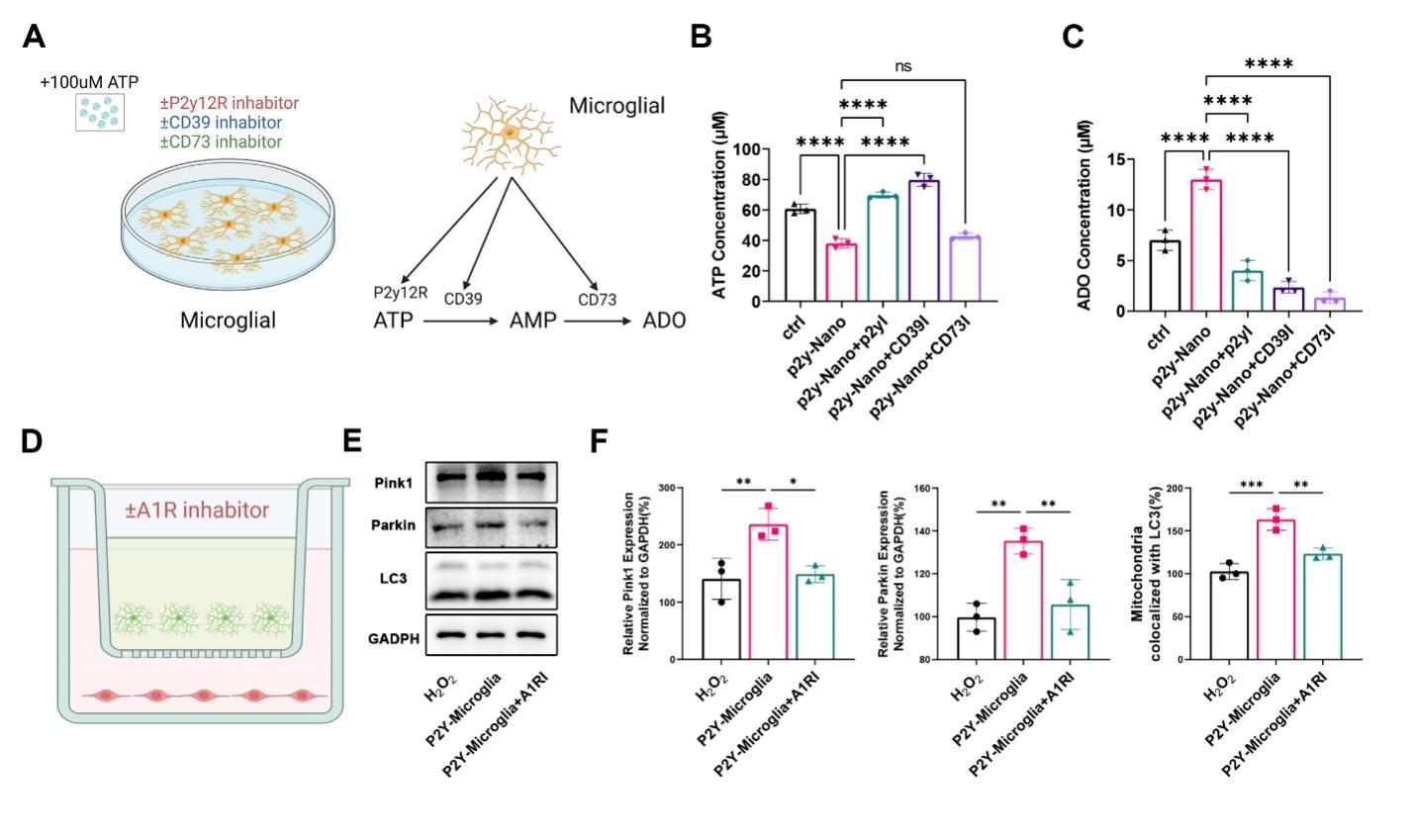
.

**Microglia promote neuronal mitophagy via ATP degradation to ADO.**

(A) Schematic representation of ATP recognition by P2Y12R in microglia and its degradation to ADO; (B-C) Quantitative analysis of the ATP and ADO concentration (samples, n=3); (D) Schematic representation of A1R inhibition in co-culture model of neurons and microglia; (B-C) Quantitative analysis of the ATP and ADO concentration (samples, n=3); (E) Western blot of Pink1/Parkin /LC3 pathway; (F) Quantitative analysis of the expression of mitophagy related proteins in (E) (samples, n=3). Data were presented as mean ± SD. Results were analyzed by One-way ANOVA. Significance: *P < 0.05, **P < 0.01, ***P < 0.001，****P < 0.0001.

Figure. S6


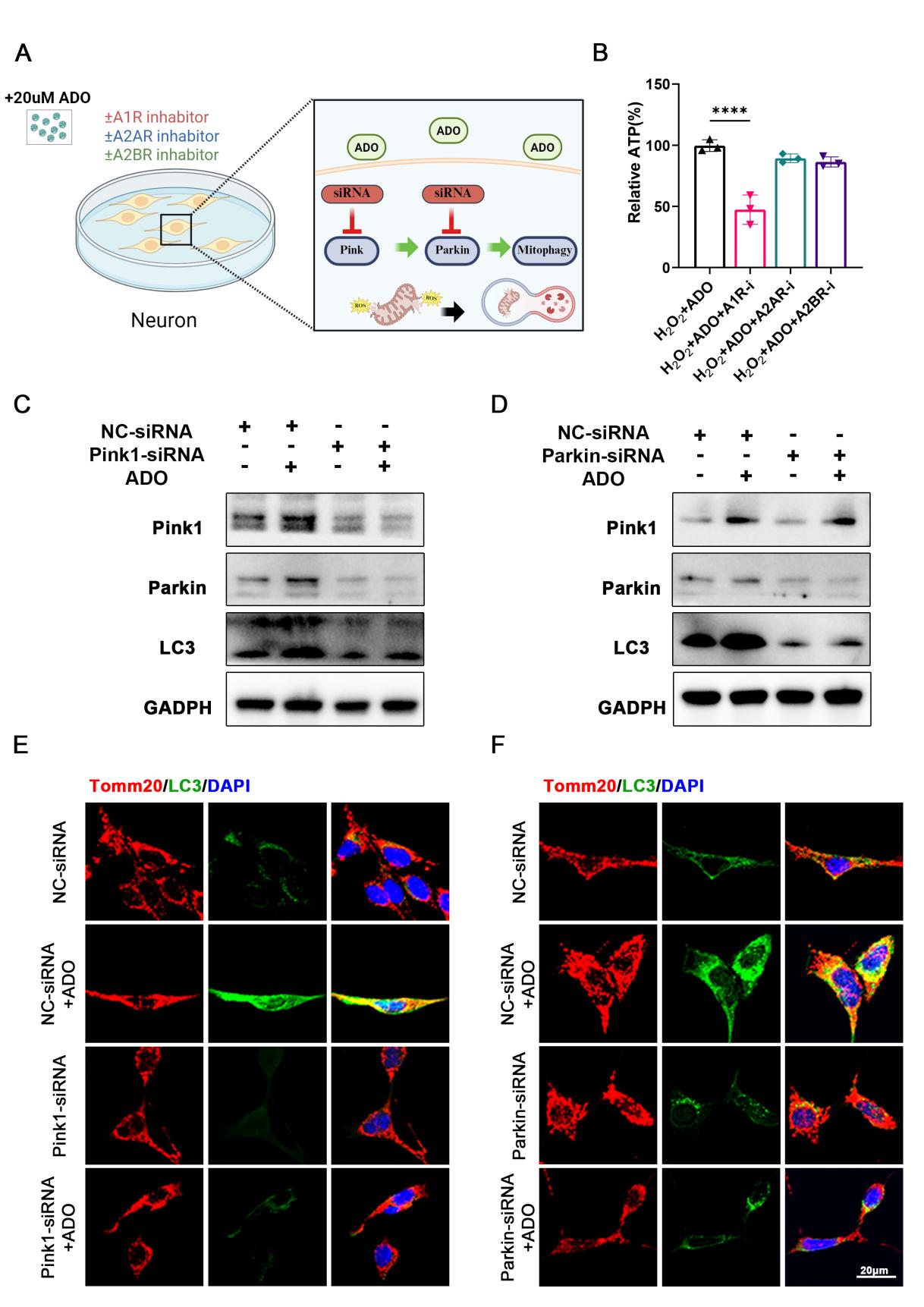
.

**ADO activates Pink1/Parkin pathway-mediated mitophagy via neuronal A1R.**

(A) Schematic of the Neuronal Monoculture Experiment; (B) Quantitative analysis of the ATP production in HT22 (samples, n=3); (C-D) Western blot of Pink1/Parkin /LC3 pathway; (E-F) Immunofluorescence of HT22 cells, lysosome and mitochondria were labeled with LC3 and Tomm20 respectively. Data were presented as mean ± SD. Results were analyzed by One-way ANOVA. Significance: ****P < 0.0001.

Figure. S7


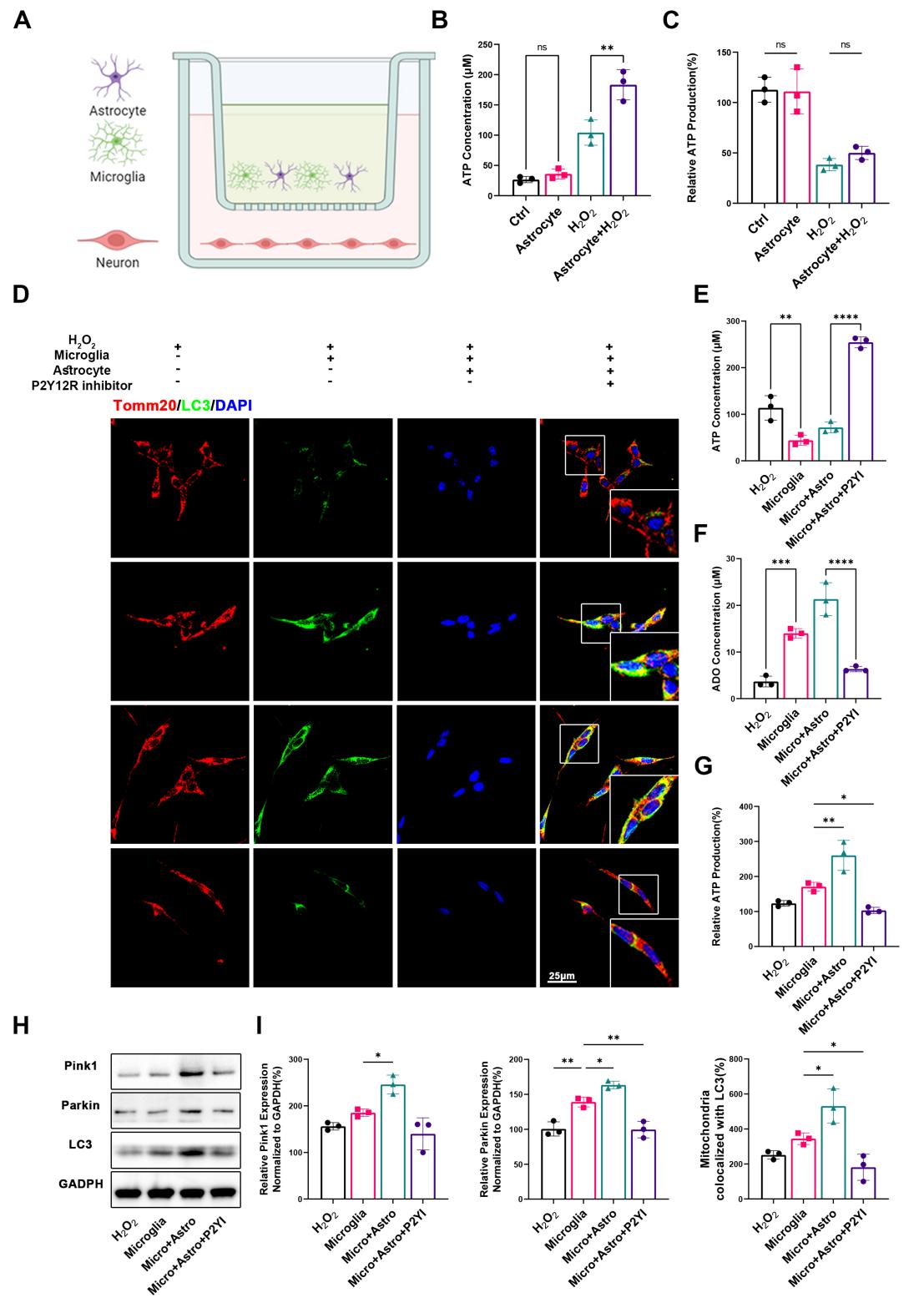
.

**ADO activates Pink1/Parkin pathway-mediated mitophagy via neuronal A1R.**

(A) Schematic of the microglia–neuron–astrocyte tri-culture system; (B) Quantitative analysis of the ATP concentration (samples, n=3); (C) Quantitative analysis of the ATP production in HT22 (samples, n=3); (D) Immunofluorescence of HT22 cells, lysosome and mitochondria were labeled with LC3 and Tomm20 respectively; (E) Quantitative analysis of the ATP concentration (samples, n=3); (F) Quantitative analysis of the ADO concentration (samples, n=3); (G) Quantitative analysis of the ATP production in HT22 (samples, n=3); (H) Western blot of Pink1/Parkin /LC3 pathway; (I) Quantitative analysis of the expression of mitophagy related proteins in (H) (samples, n=3). Data were presented as mean ± SD. Results were analyzed by One-way ANOVA. Significance: *P < 0.05, **P < 0.01, ***P < 0.001，****P < 0.0001.

Figure. S8.


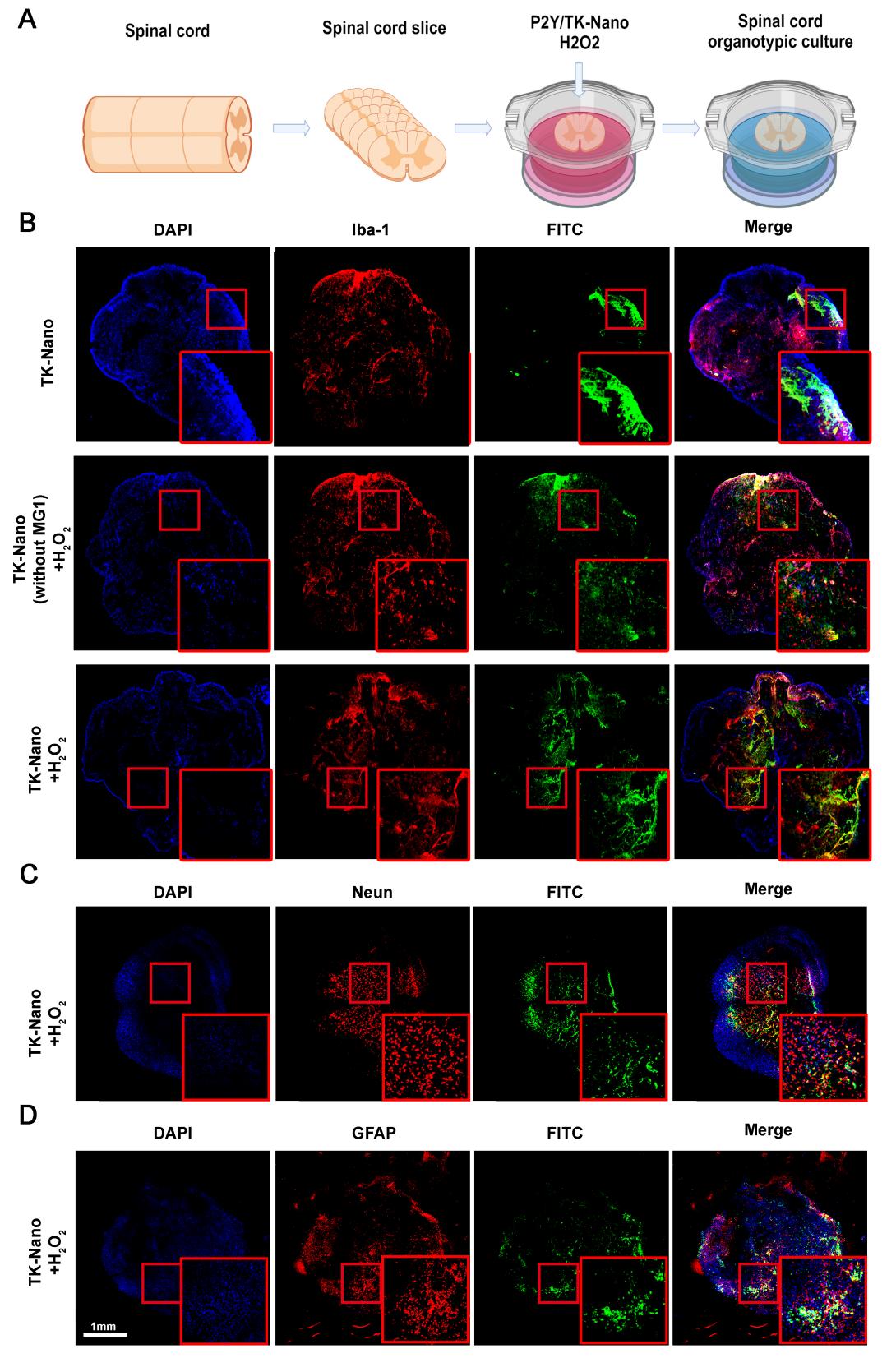


**Microglia-targeting effects of P2Y-Nano in organotypic spinal cord.**

(A) Schematic diagram of spinal organotypic experiment; (B) Immunofluorescence of spinal cord slice. Nucleus, microglia, and nanoparticles were labeled with DAPI, Iba-1, and FITC, respectively; (C) Immunofluorescence of spinal cord slice. Nucleus, neurons, and nanoparticles were labeled with DAPI, Neun, and FITC, respectively; (D) Immunofluorescence of spinal cord slice. Nucleus, astrocytes, and nanoparticles were labeled with DAPI, GFAP, and FITC, respectively

**Figure. S9.**

**
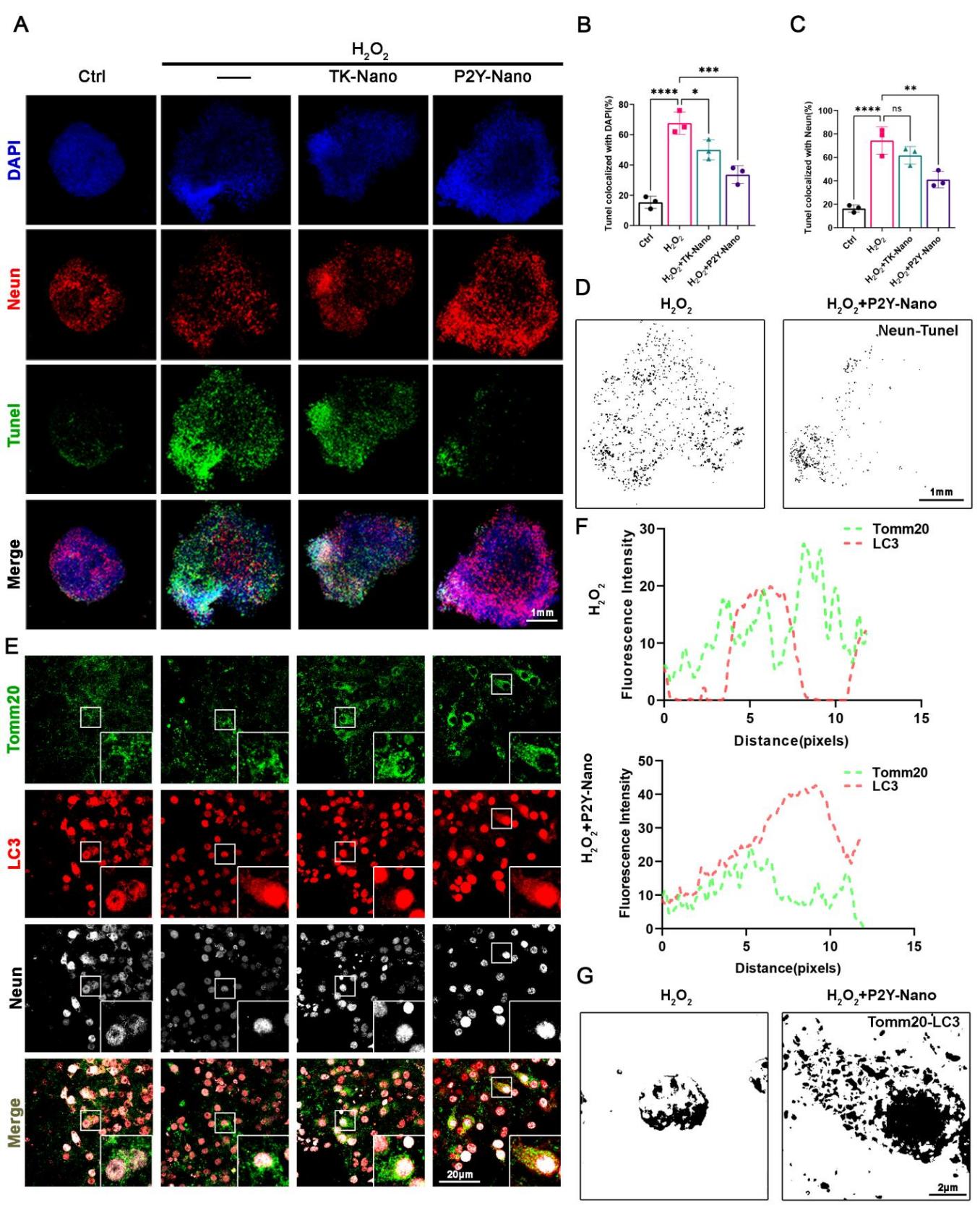
**

**Neural protection effects of P2Y-Nano in organotypic spinal cord.**

(A) Immunofluorescence of spinal cord slice. Nucleus, apoptotic cells, and neurons were labeled with DAPI, Tunel, and Neun, respectively; (B-C) Quantification of the average level of colocalization between Tunel and Neun or Tunel and DAPI in (A) (samples, n=3); (D) Co-localized Tunel and Neun in (A) ; (E) Immunofluorescence of spinal cord slice, autophagic cells, mitochondria, and neurons were labeled with LC3, Tomm20, and Neun, respectively; (F) Colocalization analysis of the fluorescence intensity of LC3 and Tomm20 in (E) ; (G) Co-localized LC3 and Tomm20 in (E). Data were presented as mean ± SD. Results were analyzed by One-way ANOVA. Significance: *P < 0.05, **P < 0.01, ***P < 0.001，****P < 0.0001, ns means no significance.

Figure. S10.


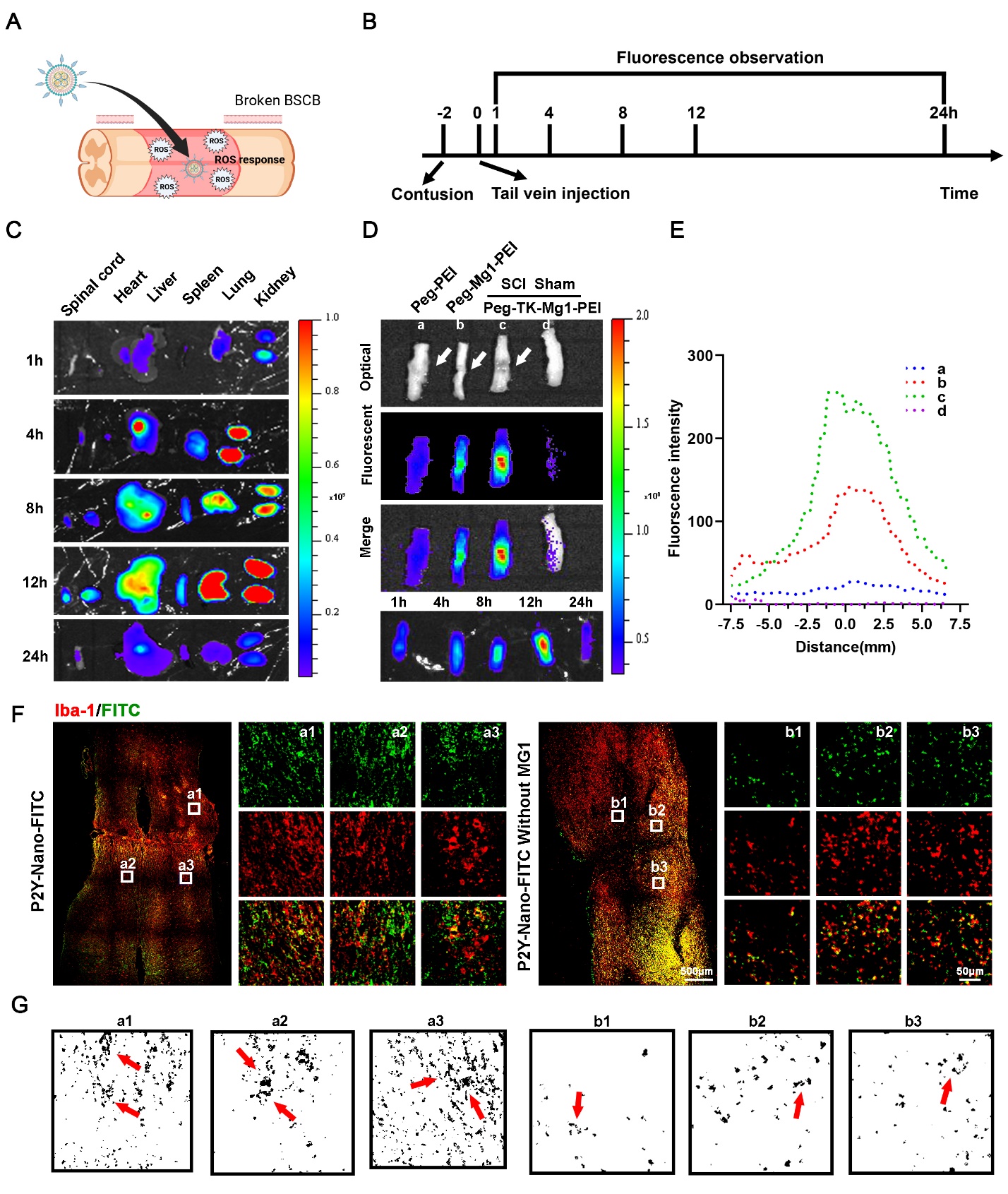


**Targeting and accumulation of P2Y-Nano in the injured spinal cord microglia.**

(A-B) Schematic diagram and experimental timeline for intravenous injection of P2Y-Nano; (C-D) Fluorescence imaging in the spinal cord and visceral organs over time of SCI mice; Quantitative analysis of fluorescence intensity in (D);(F) Immunofluorescence of spinal cord, microglia andP2Y-Nano were labeled with Iba-1and FITC, respectively;(G) Co-localized Iba-1and FITC in (F); (H) Colocalization analysis of the fluorescence intensity of Iba-1and FITC in (F)

**Figure. S11.**


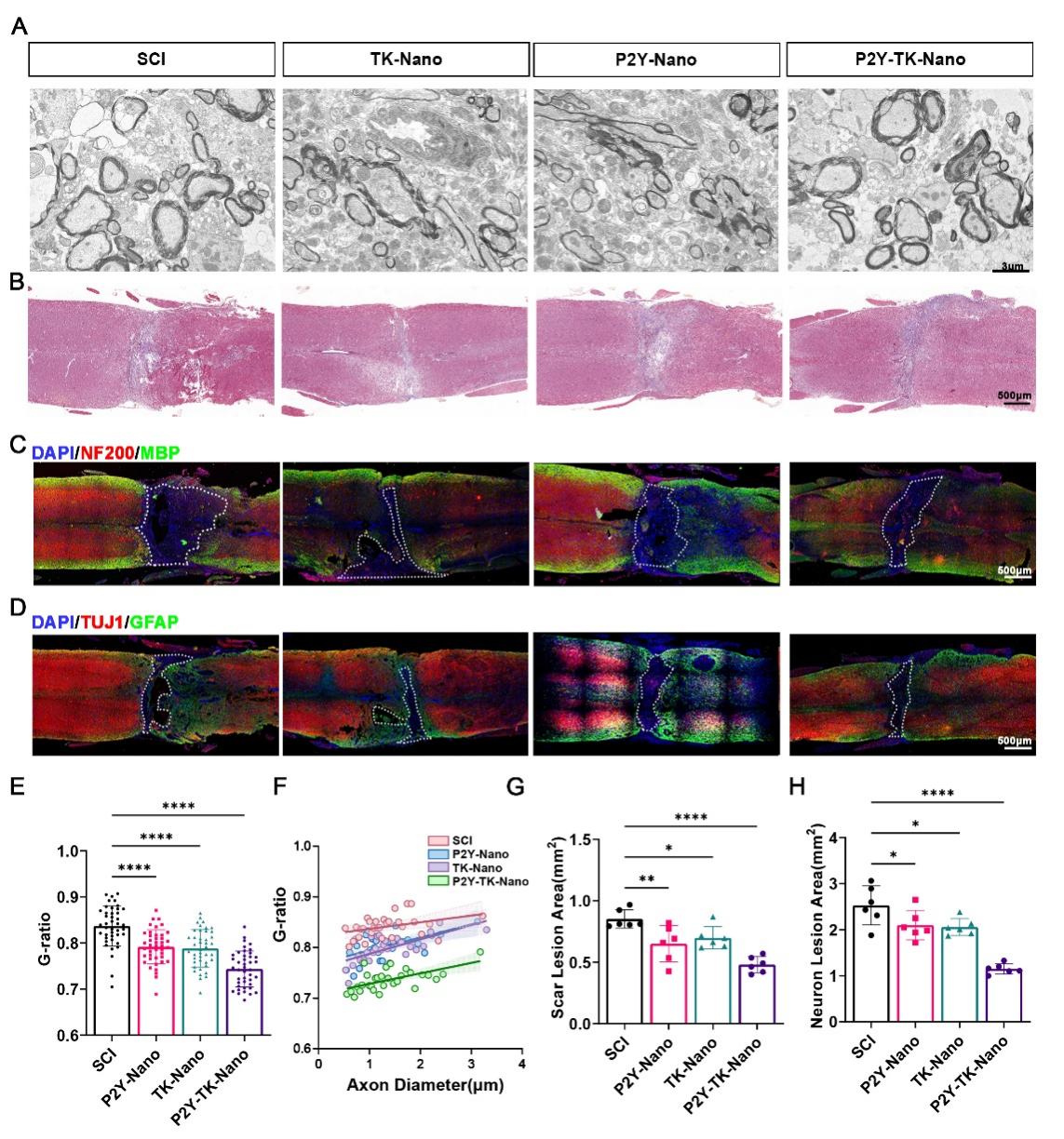


**Assessment of nerve regeneration 4-week post-SCI with P2Y-TK-Nano treatment.**

(A) Representative TEM images of myelin sheaths in SCI mice; (B) Masson Staining of the spinal cords in each group;(C-D) The immunofluorescence staining of spinal cords 4 week after injury, nerve filaments, myelin sheaths, Neuronal β-Tubulin, and astrocytes were labeled with NF200, MBP, TUJ1 and GFAP respectively; (E, F) G-ratio analysis of myelin sheaths in (A) (axons, n=30); (G,H) Quantification of the lesion area 4 week after injury in (C,D), **P < 0.01, ns means no significance. Data were presented as mean ± SD. Results were analyzed by One-way ANOVA. Significance: *P < 0.05, ***P < 0.001，****P < 0.0001.

Table S1.

PCR Primer

| Primer Name | Species | 5' -3' | Sequence |
| --- | --- | --- | --- |
| P2ry12 | Mouse | Forward | AGGGTCACAGTGCAAGAACAC |
| P2ry12 | Mouse | Reverse | TGGAACTTGCAGACTGGCAT |
| Nefh | Mouse | Forward | GCTGCTCGGTCAGATCCA |
| Nefh | Mouse | Reverse | CGGTCCAACCTCACTCGG |
| Tubb3 | Mouse | Forward | AGCGGCAACTATGTAGGGGA |
| Tubb3 | Mouse | Reverse | CAGCACCACTCTGACCAAAGATA |
| Gap43 | Mouse | Forward | AGGAGCCTAAACAAGCCGATG |
| Gap43 | Mouse | Reverse | CTTCGTCTACAGCGTCTTTCTCC |
| Syn1 | Mouse | Forward | CCCCCAGCTCAAAGCCAG |
| Syn1 | Mouse | Reverse | GAGAAGAGGCTGGCGAAAGA |
| Dlg4 | Mouse | Forward | AGCCCCAGGATATGTGAACG |
| Dlg4 | Mouse | Reverse | TCACCGATGTGTGGGTTGTC |
| Gfap | Mouse | Forward | GCGAAGAAAACCGCATCACC |
| Gfap | Mouse | Reverse | AAGGGAGAGCTGGCAGG |
| Tnf | Mouse | Forward | AGCCGATGGGTTGTACCTTG |
| Tnf | Mouse | Reverse | ATAGCAAATCGGCTGACGGT |
| IL-1B | Mouse | Forward | TGGGAAACAACAGTGGTCAGG |
| IL-1B | Mouse | Reverse | ATTAGAAACAGTCCAGCCCATACTT |

Table S2.

| **Abbreviation** | **Composition** | **Primary purpose** |
| --- | --- | --- |
| TK-Nano | PEG-TK-MG1-PEI | ROS-responsive therapeutic effect via the TK bond |
| P2Y-Nano | PEG-MG1-PEI (loading P2ry12 gene plasmid) | Therapeutic effect of P2Y12R overexpression |
| P2Y-TK-Nano | PEG-TK-MG1-PEI (loading P2ry12 gene) | Combined ROS-responsive effect + P2Y12R overexpression |
| P2ry12-Nano | PEI (loading P2ry12 gene plasmid) | In vitro-only construct for P2Y12R overexpression |
| TK-Nano-FITC | PEG-TK-MG1-PEI-FITC | Uptake validation for TK-Nano |
| P2Y-TK-Nano-FITC | PEG-TK-MG1-PEI (loading P2ry12 gene)-FITC | Uptake validation for P2Y-TK-Nano |
| TK-Nano-FITC (without MG1) | PEG-TK-PEI-FITC | Validating the targeting contribution of MG1 |
